# Selectively advantageous instability in biotic and pre-biotic systems and implications for evolution and aging

**DOI:** 10.3389/fragi.2024.1376060

**Published:** 2024-05-16

**Authors:** John Tower

**Affiliations:** Molecular and Computational Biology Section, Department of Biological Sciences, University of Southern California, Los Angeles, CA, United States

**Keywords:** evolution of aging, replicator, mitochondria, grandmother hypothesis, complexity, autopoiesis, cellular automata, sex differences

## Abstract

Rules of biology typically involve conservation of resources. For example, common patterns such as hexagons and logarithmic spirals require minimal materials, and scaling laws involve conservation of energy. Here a relationship with the opposite theme is discussed, which is the selectively advantageous instability (SAI) of one or more components of a replicating system, such as the cell. By increasing the complexity of the system, SAI can have benefits in addition to the generation of energy or the mobilization of building blocks. SAI involves a potential cost to the replicating system for the materials and/or energy required to create the unstable component, and in some cases, the energy required for its active degradation. SAI is well-studied in cells. Short-lived transcription and signaling factors enable a rapid response to a changing environment, and turnover is critical for replacement of damaged macromolecules. The minimal gene set for a viable cell includes proteases and a nuclease, suggesting SAI is essential for life. SAI promotes genetic diversity in several ways. Toxin/antitoxin systems promote maintenance of genes, and SAI of mitochondria facilitates uniparental transmission. By creating two distinct states, subject to different selective pressures, SAI can maintain genetic diversity. SAI of components of synthetic replicators favors replicator cycling, promoting emergence of replicators with increased complexity. Both classical and recent computer modeling of replicators reveals SAI. SAI may be involved at additional levels of biological organization. In summary, SAI promotes replicator genetic diversity and reproductive fitness, and may promote aging through loss of resources and maintenance of deleterious alleles.

## 1 Introduction

At first inspection, it might seem that the most efficient and competitive way for cells to grow and divide would be to make all the cell components as stable as possible, thereby maximizing the biomass available to create new cells. However, decades of cell research tells us that is not the case. There are several well-studied mechanisms where it is beneficial for cell components to be short-lived. For example, many transcription factors and signaling factors are short-lived, enabling the cell to rapidly change its physiology in response to environmental stress. Because the instability of these cell components favors cell survival and subsequent division, it indicates these components exhibit selectively advantageous instability (SAI). Other examples of SAI are less well-studied, and examples of SAI have generally not been compared for possible similar features. The goal of this review is to survey a variety of well-known and proposed examples of SAI, and explore a minimal set of models that might define SAI across multiple levels of biological organization.

## 2 Rules of biology typically involve conservation of resources

Organizing principles or rules that function at multiple levels of organization are relatively rare in biology (Dhar and Giuliani, 2010; Rull, 2022). Most of the known biological rules can be interpreted as having a common theme, which is the conservation of components of the system (Hodapp et al., 2019; Robert Burger et al., 2021). Examples include common shapes, such as hexagons and logarithmic spirals, as well as scaling laws.

**FIGURE 1:**
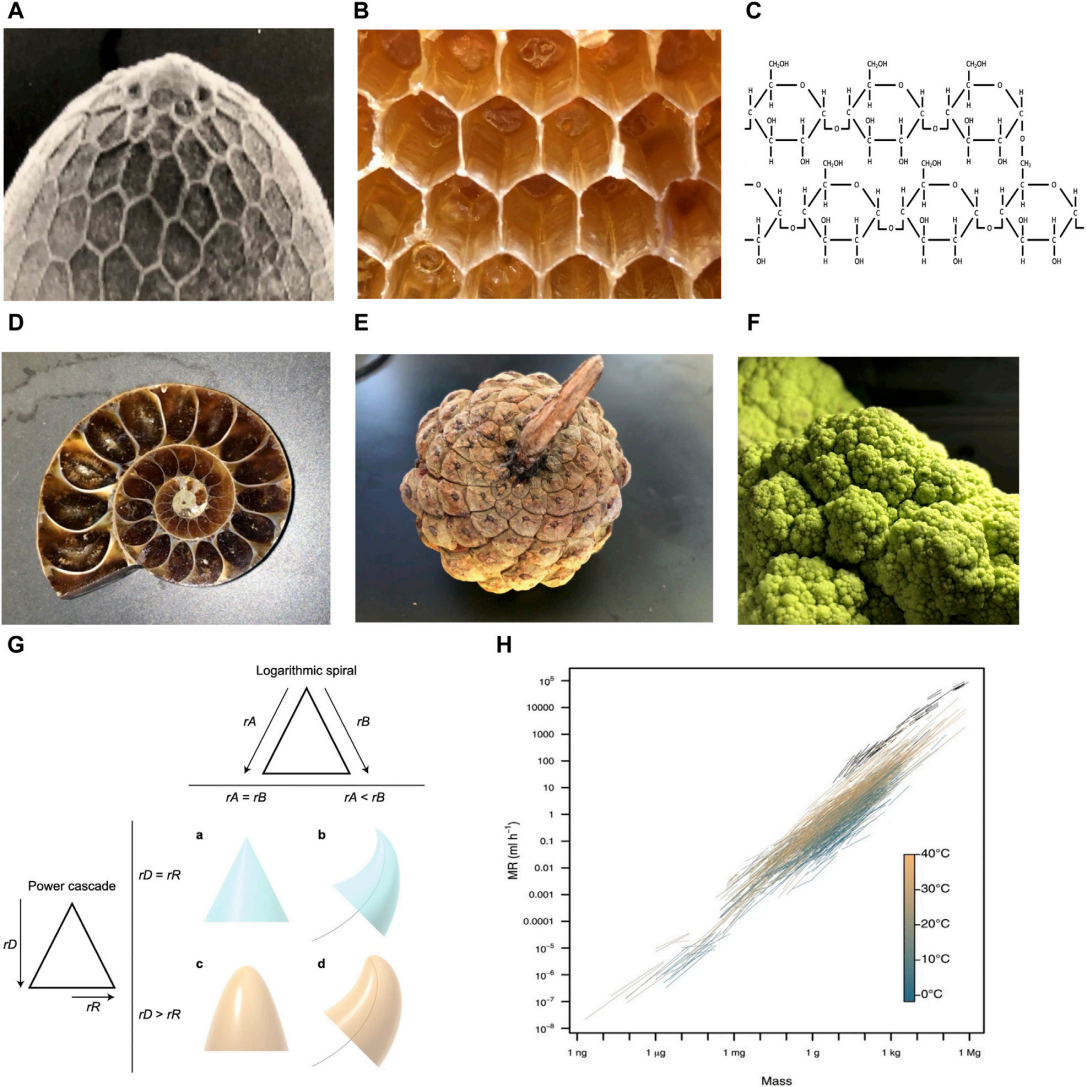
Examples of organizing principles observed across multiple levels of biological organization. Photo credit John Tower. **(A–C)** Hexagons. **(A)** *Drosophila* eggshell. **(B)** Honeycomb. **(C)** Glycogen. **(D–F)** Logarithmic spirals and self-repeating patterns. **(D)** Ammonite fossil. **(E)** Pinecone. **(F)** Romanesco broccoli. **(G)** Power cone model. Logarithmic spiral (top): if the rates of growth of the two sides of the structure (rA and rB) are equal, a symmetrical structure such as a cone is produced **(A)**. If the rate of growth on one side is lower (e.g., rA < rB), then the structure curves to follow a logarithmic spiral (black curved line) **(B)**. Power cascade (left): when the power growth rate of the distance from the tip (rD) is equal to the growth rate of the radius (rR), then a cone is produced **(A)**. When rR is less than rD, a power cone is generated **(C)**. Here, rD = 2rR, generating a paraboloid. Both of these inequalities in growth rates can be combined to form a power cone curving along a logarithmic spiral, or a power spiral **(D)**. Figure reproduced without changes from Evans *et al.* BMC Biology (2021) 19:58. **(H)** Scaling of metabolic rate (MR) with body mass. Black lines depict intraspecific scaling relationships for endotherms (birds and mammals). Colored lines depict intraspecific scaling relationships for ectotherms (fishes, amphibians, reptiles, and invertebrates), which are colored by measurement temperature from 1.8°C to 40°C. Figure reproduced without changes from White et al. Science (2022) 377:834–839.

Hexagons are observed at multiple levels of biological organization, including the *Drosophila melanogaster* eggshell (Figure 1A), the honeycomb (Figure 1B), and the 6-membered rings of the carbohydrate glycogen (Figure 1C). This conserved pattern is thought to result because hexagons require the least amount of material to cover a surface and are energetically stable (Hales, 2001). In addition, modeling indicates that monomers shaped like hexagons can more rapidly self-assemble into higher-order structures than can monomers shaped like squares or triangles (Gartner and Frey, 2024). Because hexagons can spontaneously form under appropriate conditions, they require minimal information to be encoded in the organism.

Logarithmic spirals, including the golden spiral and Fibonacci spiral, are observed at multiple levels of biological organization, including mollusk shells (Figure 1D), pinecones (Figure 1E), and Romanesco broccoli (Figure 1F). Self-similar structures, including logarithmic spirals, are thought to be the most economical way to grow the size of a structure, without changing shape or destroying existing structure (Cardoso et al., 2020; Wada et al., 2020). In addition, these structures also require minimal information to be encoded in the organism. For example, Evans et al. showed that a large variety of biological structures, including teeth, spiral shells, beaks and claws can be described by a simple relationship called the power cone, which is generated when the radial power growth rate is unequal to the length power growth rate (Figure 1G) (Evans et al., 2021).

Scaling laws can also be interpreted in terms of conservation of materials and energy (West and Brown, 2004; Kitazoe et al., 2019). One commonly observed relationship in biology is allometric scaling, where a biological variable Y is related to the mass of the organisms (M), by the power law, Y = Y_0_M^b^, where b is the allometric exponent. For example, the basal metabolic rate (BMR) of most organisms can be described by a power law with b ranging from 3/4 to 2/3 (da Silva et al., 2006). This relationship has been suggested to result from physical constraints such as the need to dissipate heat, and constraints on surface area/volume ratios created by requirements for the movement of nutrients and waste products. Other studies suggest a related model in which allometric scaling results from selection to optimize metabolism, growth and lifetime reproduction (White et al., 2022; White and Marshall, 2023). The absence of organisms that vary significantly from the line relating metabolic rate to body mass indicates selection and/or constraints to minimize energy expenditure (Figure 1H).

## 3 Selectively advantageous instability

**FIGURE 2:**
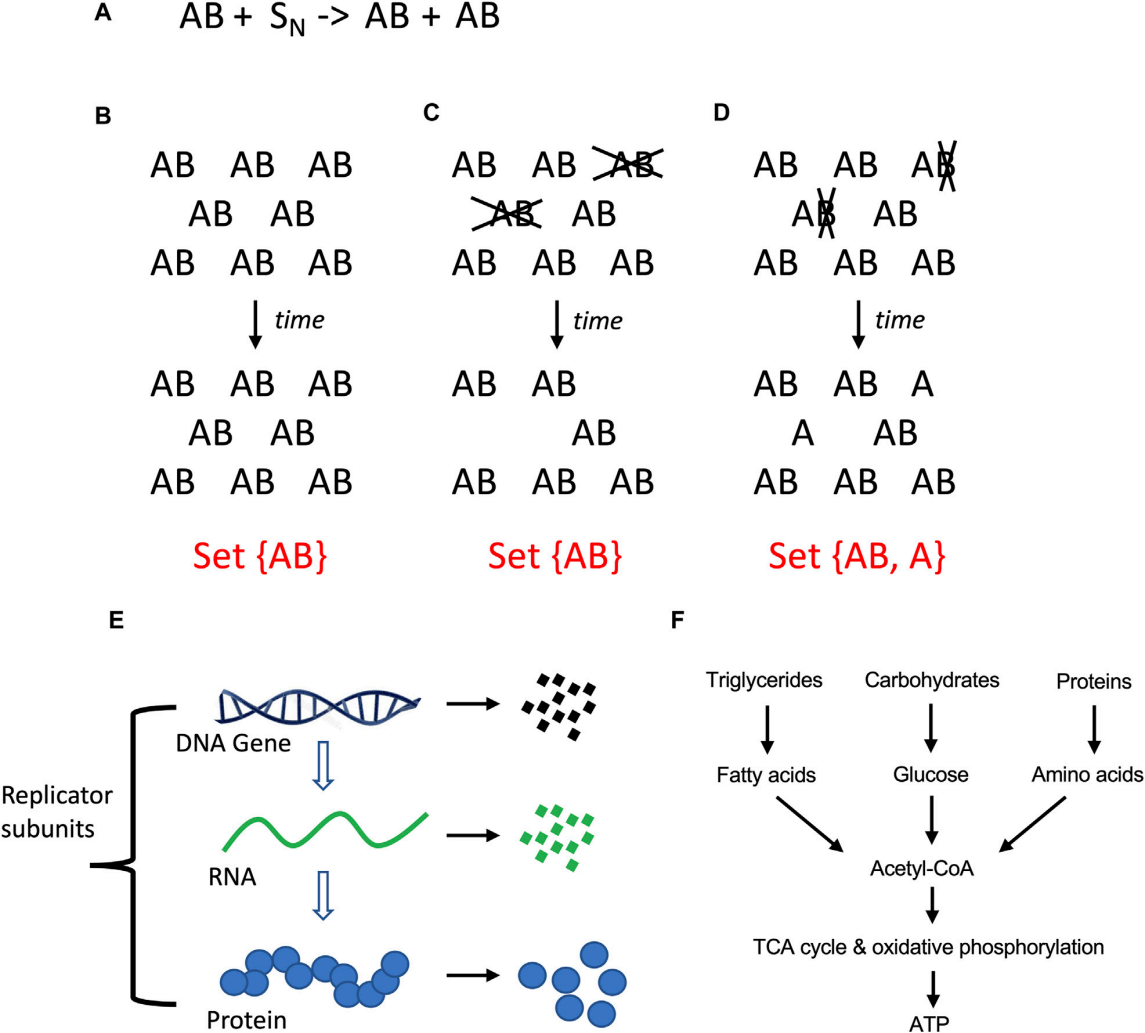
Molecular replicators and selectively advantageous instability (SAI). **(A)** Equation for two-subunit replicator. The two-subunit replicator AB utilizes N number of substrate molecules (S) to generate a new copy of AB. **(A)** Stability of AB. If AB is stable, replication of AB with time produces more AB replicators, yielding a set with only one member. **(C)** Instability of AB. If the A and B subunits of the AB replicator are unstable (indicated in gray font), and degraded at the same time, this leads to a reduced population of AB replicators, again yielding a set with only one member. **(D)** Instability of **(B)**. If the B subunit is less stable than A subunit (indicated in gray font), this leads to a reduced population of AB replicators and free A subunits; this represents a more complex system because it yields a set with two distinct members. **(E)** Instability is defined here as the physical degradation of the replicator subunit through breakage of bonds. **(F)** Catabolic metabolism. Complex polymeric macromolecules are degraded to monomeric building blocks, and these products can be further degraded to liberate energy.

Here a relationship with the opposite theme is discussed, which is the selectively advantageous instability (SAI) of one or more components of a replicating system. When two genes or subunits (A and B) collaborate to create a replicator (AB), it may be selectively advantageous for B to have a shorter half-life than A, i.e., to be unstable. This is because SAI increases the complexity of the system, and this increased complexity has potential benefits (Tower, 2006). When a hypothetical AB replicator replicates, it produces new copies of AB, yielding a set with only one member, set {AB} (Figures 2A,B). If AB is unstable, and A and B are degraded at the same time, this also results in a set with only one member, set {AB} (Figure 2C). However, if B is less stable than A, this results in a set with two members, set {AB, A} (Figure 2D). Because a set with two members is more complex than a set with one member, SAI inherently increases the complexity of the system. At the same time, SAI involves a potential cost to the replicating system for the materials and energy required to create the unstable component, and in some cases, the energy required for its active degradation. Previous modeling of hypothetical AB replicators supports the idea that the instability of B becomes selectively advantageous when the benefits of increased complexity outweigh the cost in terms of replicator proliferation (Li et al., 2018; Qu et al., 2021).

SAI can be hypothesized to function at multiple levels of biological organization. At the level of molecular replicators, A and B are both genes and subunits. At the level of the cellular replicator, A and B can be genes, or other cell subunits such as nucleus, mitochondria, cell membrane, proteins or RNAs. At the level of the organism, A and B can be genes, cell types, or tissues. And finally, at the level of social structures, A and B might be nodes or links. To define SAI requires that some additional terms be operationally defined.

### 3.1 Instability

In the context of SAI, instability is defined as the relative propensity of the subunit to undergo a physical disintegration of its structure, either spontaneously, or through the action of degradative agent such as an enzyme. For polymers such as DNA, RNA and proteins, this involves degradation through hydrolytic cleavage into fragments and/or monomers, and possible further degradation of the monomers (Figure 2E). This type of instability involves breakage of covalent bonds, and is referred to here as degradation (Table 1). A propensity for abnormal folding or denaturation of proteins is often described as folding instability, and indeed abnormal folding is often a signal that targets a protein for degradation (Slominska-Wojewodzka and Sandvig, 2015; Balchin et al., 2016; Sala et al., 2017). Therefore, folding instability is distinct from degradation, but can sometimes serve as a signal for degradation. Signals for degradation and determinants of half-life are encoded in the primary structure for proteins and other macromolecules (Slominska-Wojewodzka and Sandvig, 2015; Pownall et al., 2016; Varshavsky, 2019).

**TABLE 1:** SAI types and selected examples.

SAI types	Energy requirements	Selected examples	Models
**Degradation (covalent bond breakage)**	Degradation that releases building blocks and generates energy	Catabolism of lipids, carbohydrates, and AAs	CM1 (catabolic metabolism 1)
	Degradation that releases building blocks and requires energy	Protein breakdown to AAs	CM2 (catabolic metabolism 2)
		Otto replicator cycling	
	Degradation with benefits in addition to release of energy or building blocks	**Removal of damage**	3
		Basal turnover of protein, lipids and RNA	
		Targeted degradation of damaged proteins and RNAs	
		Mitophagy	
		PCD in multicellular organisms	
		Germ/line soma distinction	
		**Adaptation to environment**	2
		Stress response - short-lived transcription factors and mRNAs	
		TA systems	
		Telomeres	
		Cell division factors and cyclins	
		Fletcher replicator micelle size and abundance	
		Autopoiesis boundary size model	
		Langton loop connector and arm models	
		**Maintenance of genetic diversity**	2
		TA systems	
		Uniparental mitochondrial transmission	
		**Beneficial functions of free A**	1
		Grandmother hypothesis	
		Otto replicator complexity (?)	
**Static assembly disruption**	Requires energy to disrupt	**Adaptation to environment**	2
		HSF trimers	
		Nucleosomes	
**Dynamic assembly**	Requires energy to maintain	**Adaptation to environment**	2
		Actin filaments	
		Microtubules	
		Otto replicator	
		Fletcher replicator	
		Autopoiesis boundary	
		The cell	

### 3.2 Degradation and metabolism

Metabolism has been defined as the sum of chemical reactions that take place in the cell to maintain life (Judge and Dodd, 2020). Metabolism can be generally divided into the synthesis of complex molecules (anabolic metabolism), which requires energy, and the degradation of complex molecules (catabolic metabolism), which can generate energy (Figure 2F). The major sources of energy in the heterotrophic animal cell include fatty acids, glucose and amino acids. In the fed state, the animal cell derives the majority of energy from breakdown of dietary triglycerides and carbohydrates, with a relatively smaller contribution from breakdown of dietary proteins. However, during fasting, the cell and organism can mobilize protein stores for energy. This includes degradation of ribosomes and muscle structural proteins to amino acids, which are then used for gluconeogenesis (Fryburg et al., 1990). Catabolic metabolism that generates energy is referred to here as catabolic metabolism type 1 (CM1; Table 1).

In addition to the generation of energy, degradation of macromolecules in the cell can be beneficial by providing building blocks for the synthesis of new macromolecules with different structure or sequence. For example, the mobilization of amino acids from muscle protein stores during fasting, as mentioned above, can be used to support new protein synthesis. Similarly, many developing organisms generate abundant “storage proteins,” which function as a reservoir of amino acids for future protein synthesis needs (Haunerland, 1996; Muntz, 1998; He et al., 2021). Notably, these degradation pathways require some energy, for example, the ATP-dependent unfoldases and proteases involved in protein degradation (Balchin et al., 2016). Catabolic metabolism that liberates building blocks and requires energy is referred to here as catabolic metabolism type 2 (CM2; Table 1).

The degradation of subunits associated with SAI is expected to typically proceed through the same catabolic metabolism pathways the cell normally uses to produce energy and/or mobilize building blocks. However, the selective advantage(s) of subunit degradation in SAI are hypothesized to include benefits in addition to the generation of energy or mobilization of building blocks.

### 3.3 Hydrolysis and enzymatic breakdown

In the cell, the degradation of polymeric macromolecules involves cleavage of covalent bonds by enzymes, including nucleases, proteases and hydrolases (Saftig, 2006; Kaushik and Cuervo, 2018; Rousseau and Bertolotti, 2018). The majority of cellular macromolecules exhibit little if any spontaneous hydrolysis due to the aqueous environment (Wolfenden and Yuan, 2008; Gates, 2009; Butre et al., 2015). One partial exception to this rule is the glycosidic bonds that connect the nucleobases to the phosphodiester backbone of RNA and DNA, which are relatively susceptible to spontaneous hydrolysis. This spontaneous hydrolysis is estimated to yield ∼10,000 abasic DNA sites per cell per day (Lindahl and Nyberg, 1972). Spontaneous hydrolysis might be more relevant to pre-biotic and acellular replicators, as discussed below.

### 3.4 Selective advantage

Selectively advantageous instability (SAI) is defined as instability of a subunit that increases the replicative fitness of the replicator and/or the replicator lineage. For the purposes of the present modeling, replicative fitness is measured by the number of new progeny replicators produced per unit time by an individual replicator or a group of collaborating replicators.

### 3.5 Self-assembly

Two additional types of instability are also briefly discussed, which are dissociation of static assemblies and dynamic assemblies (Table 1). Self-assembly is mediated by non-covalent interactions, and is generally defined as the autonomous organization of pre-existing individual components into ordered patterns or structures, based upon information encoded in the individual components, such as shape, charge, and hydrophobicity (Whitesides and Grzybowski, 2002). Self-assembly can be generally divided into two forms, static self-assembly and dynamic (dissipative) self-assembly. Static self-assembly is driven by the gain in free energy when the assembled structure represents a local or global minimum in the free energy landscape, resulting in a structure that is at equilibrium and stable with time (Fialkowski et al., 2006; Mattia and Otto, 2015). Examples of static self-assembly include crystals, lipid micelles (Alexandridis et al., 1994), and multi-subunit complexes such as HSF trimers (Kmiecik et al., 2020), and the nucleosome (Imbalzano et al., 1996; Langst and Becker, 2004; Becker and Workman, 2013). Disruption of static assemblies requires energy.

Dynamic self-assembly requires a continuous source of energy in order to generate and maintain a functional structure that is far from equilibrium, and this energy is dissipated to the environment in a form such as heat (Fialkowski et al., 2006; Qian, 2006; Wagner and Pross, 2011; Maiti et al., 2016; Danger et al., 2020). Examples include actin filaments (Wear et al., 2000; Plastino and Blanchoin, 2018) and microtubules (Mitchison and Kirschner, 1984; Horio and Murata, 2014). Conceivably, the energy-dependent disruption of static assemblies, and the energy-dependent maintenance of dynamic assemblies might be interpreted as special types of SAI (Table 1).

## 4 SAI may link evolution and aging

**FIGURE 3:**
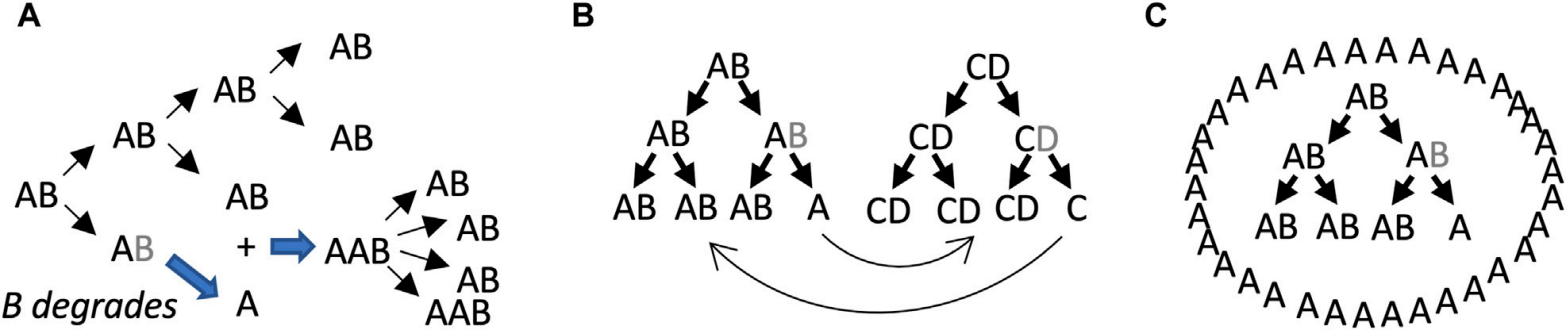
SAI model 1, beneficial function of free A subunits. In these models, AB is capable of self-replication, B is unstable, and free A cannot self-replicate. Multiple scenarios can be envisioned where the free A subunit provides a selective advantage to the replicator lineage. **(A)** Stimulatory effect of free A subunit on replication. The B subunit degrades, leading to free A subunits. The free A subunits then bind to AB replicators and stimulate their activity. Assumptions include unlimited substrate, lack of product inhibition, and instantaneous binding of A to AB. Under these conditions, both stochastic and deterministic rate equation modeling indicated that instability of B becomes favorable when replication activity of AAB is 3–8 times the replicator activity of AB. **(B)** Collaborating/cross-regulatory replicators. The free A subunit from an AB replicator could stimulate (or inhibit) the activity of a CD replicator, and in turn the free C subunit from the CD replicator could stimulate (or inhibit) the AB replicator. **(C)** Formation of a beneficial structure by free A.

One potential significance of SAI is that it may be relevant to evolution and aging. It has been proposed that the evolution of increased complexity, as measured by the number of fundamental components, is selectively advantageous by allowing a greater division of labor (Bonner, 2004; Yaeger, 2009). As discussed above, SAI increases the complexity of the system by increasing the number of distinct components (Figure 2D). Aging has proven to be difficult to define, but most definitions include an increased chance of death with age, and decreased reproductive fitness with age (Tower, 2009; Flatt, 2012). A loss of subunit B could prevent further replication, which represents an age-dependent loss of reproductive fitness for that individual replicator (see model 1, Figure 3A). A leading model for the genetics of aging in all organisms is antagonistic pleiotropy, which states that a gene can be beneficial in one context, and detrimental in another context (Williams, 1957). If there is selection for degradation of the gene in its detrimental context, then antagonistic pleiotropy may lead to SAI (Qu et al., 2021). As discussed below, SAI facilitates the maintenance of genetic diversity, and this is expected to include deleterious alleles that contribute to aging. Finally, because energy and materials are required to create subunit B, which is then lost due to instability, SAI can create a cost for the replicator in terms of energy and/or materials, and this cost might be interpreted in terms of promoting aging.

## 5 SAI in the context of evolution of life and replicators

Life has proven to be difficult to define, however, the ability to replicate and undergo Darwinian evolution are typically considered to be key features (Cleland and Chyba, 2002; Trifonov 2011; Higgs, 2017; Takeuchi et al., 2017). The basic unit of life is the cell, which can replicate and undergo Darwinian evolution, as well as carry out metabolism and store the information required to direct replication. In the cell, chromosomal genes composed of DNA encode multi-subunit protein/RNA complexes that catalyze the replication of the chromosome and cell (Sclafani and Holzen, 2007; Hunt et al., 2011).

It has been hypothesized that early stages in the evolution of life involved self-replicating macromolecules called replicators, which can be considered genes capable of self-replication (Dawkins, 1976; Kauffman, 1993; Joyce, 2002; Kauffman, 2011). The evolutionarily-conserved role of RNA molecules in fundamental cellular processes, and the discovery that RNAs can act as enzymes (ribozymes), suggests that the ancestral replicators may have been composed of RNA (Gilbert, 1986; Joyce, 2002). Consistent with this idea, RNA polymerase ribozymes have been created that can synthesize functional, non-self RNA molecules using an RNA template (Horning and Joyce, 2016). However, no single RNA polymerase ribozyme has yet been identified that is capable of complete self-replication. Other possible chemical structures for early replicators include polypeptides, other polymers, and lipid micelles (Kauffman, 1993; Kosikova and Philp, 2017; Morrow et al., 2019; Piette and Heddle, 2020; Yang et al., 2021).

The cell replicator is surrounded by a lipid membrane that helps retain molecules within the cell to support replication. Molecular replicators functioning at early stages of evolution might have been free in solution, such that the individual replicator lineage was the unit of selection, or perhaps more likely, enclosed in proto-cell membranes such that groups of collaborating replicators were the unit of selection (Lloyd, 2020). It has been suggested that the evolution of the first genomes might require a collaboration, where some replicators reduce their own replication rate to help copy others (Eigen and Schuster, 1978; Szostak et al., 2016; Levin and West, 2017), and this might be favored by inclusion of the collaborating replicators within a proto-cell membrane. The presence of an enclosing membrane is a defining characteristic of the cell, and has often been suggested to be essential for life.

## 6 Theoretical AB replicators suggest three basic models for SAI

The conceptual simplicity of molecular replicators facilitates the development of three basic SAI models that might then be applied to SAI at multiple levels of biological organization. The hypothetical replicator (AB) is considered, which is composed of only two subunits, the stable A subunit and the relatively unstable B subunit.

### 6.1 Model 1: beneficial effects of free A

The model 1 involves the beneficial effects of free A subunits to increase the replicative fitness of intact AB replicators (Figure 3). In this model, the loss of B leaves the A subunit unable to replicate, and therefore SAI is causing reproductive senescence of that replicator. The benefit of B instability is to sibling and/or progeny replicators. In the first scenario, free A directly binds to AB replicators and stimulates their activity (Figure 3A). Assumptions include unlimited substrate, lack of product inhibition, and instantaneous binding of A to AB. Under these conditions, both stochastic modelling and deterministic modeling using rate equations indicated that instability of B is favorable when replication activity of AAB is 3–8 times greater than the replicator activity of AB (Li et al., 2018; Qu et al., 2021). Model 1 might also enable regulatory interactions between different replicators. For example, the free A subunit derived from the AB replicator might stimulate (or inhibit) a hypothetical CD replicator, and in turn, a free C subunit derived from the CD replicator might stimulate (or inhibit) the AB replicator (Figure 3B).

It is conceivable that early replicators might have existed free in solution, such that the individual replicator or replicator lineage was the unit of selection (Lewontin, 1970). However, it is perhaps more likely that early replicators or groups of collaborating replicators were surrounded by a proto-cell membrane, such that the proto-cell was the unit of selection (Griffiths, 2007; Joyce and Szostak, 2018; Lloyd, 2020; Zhang et al., 2023). Another way that free A might stimulate AB replicators is by creating a beneficial structure, such as a surrounding proto-cell membrane, that might help retain materials and collaborating replicators, and/or protect against predators (Figure 3C). Generation of a surrounding membrane by free A is reminiscent of the classical chemoton and autopoiesis models for the early cell (Cornish-Bowden and Cardenas, 2020).

### 6.2 Model 2: genetic diversity and adaptation to the environment

**FIGURE 4:**
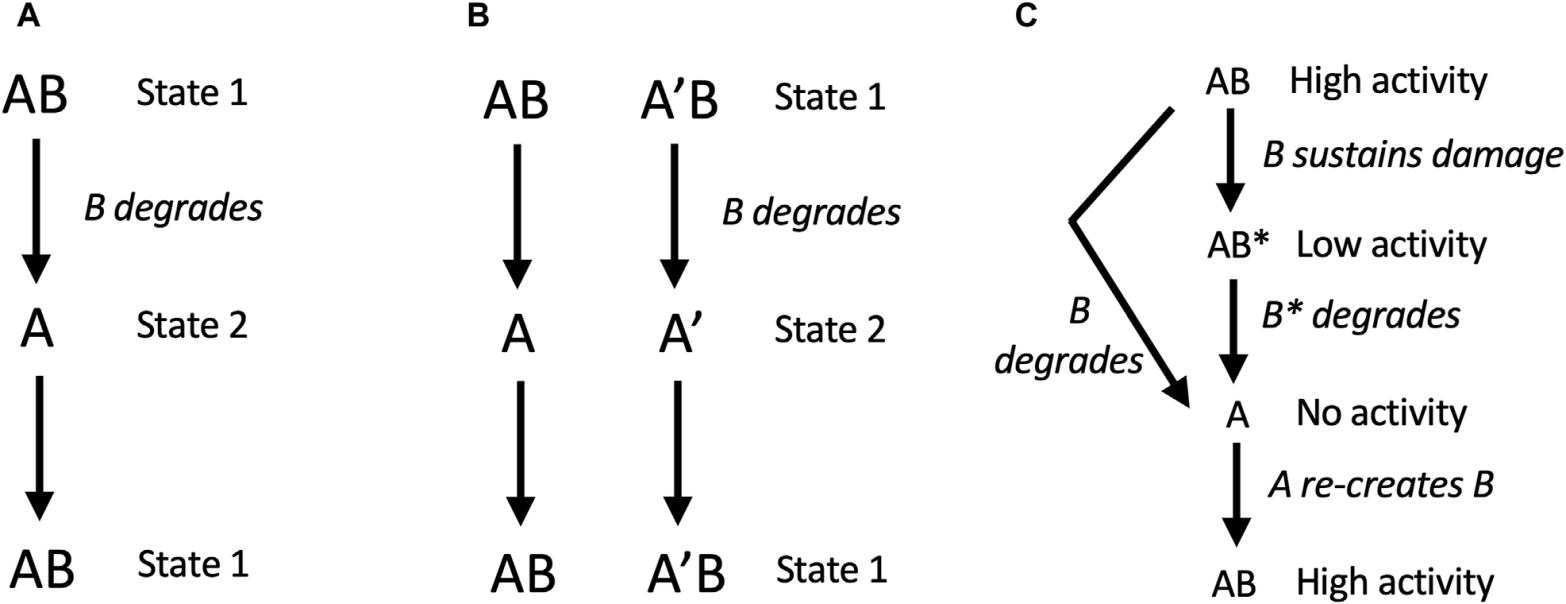
SAI promotes adaptation to environment, genetic diversity, and removal of damage. In these models, AB is capable of self-replication, B is unstable, free A cannot self-replicates, but free A can generate a new B subunit. **(A, B)** SAI model 2, adaptation to environment and maintenance of genetic diversity. **(A)** Adaptation to a changing environment. Degradation of B creates two states for the replicator: in state 1, B is present, and in state 2, B is absent. If state 1 and state 2 differently respond to the environment, for example, if A is better at function or survival in one environment, and AB is better at function or survival in another environment, then SAI can enable adaptation to a changing environment. In this model, state 1 and state 2 could represent stages in the replication process, such as stages of the cell division cycle. **(B)** Maintenance of genetic diversity. During replication of the AB replicator, the A subunit can undergo mutation to A’. If the environment selects for A in the presence of B, and selects for A’ in the absence of B (or *vice versa*), this will tend to maintain both A and A’ in the population. The same mechanism applies to the maintenance of B genetic diversity. **(C)** SAI model 3, removal of damage. B sustains damage and is replaced by a new B. In this model, B sustains damage (B*) which reduces the activity of the AB replicator. Instability of B leads to free A subunit, and A is able to generate a new B.

In model 2, free A can generate a new B subunit. Here, degradation of B creates two states for the replicator: in state 1, B is present, and in state 2, B is absent. If state 1 and state 2 differently respond to the environment, for example, if A is better at function or survival in one environment, and AB is better at function or survival in another environment, then SAI can enable adaptation to a changing environment (Figure 4A). For example, B might represent an unstable transcription factor whose presence or absence allows the cell to rapidly adapt to a new environment. Similarly, B might be a replicator component that is generated during the process of replication, that must then be degraded to complete the replication process. Model 2 also promotes the maintenance of genetic diversity. For example, during replication of the AB replicator, the A subunit can undergo mutation to A’. If two environments differently select for A *versus* A’, this will tend to maintain both A and A’ in the population, and enable adaptation to changing environment (Figure 4B). The same mechanism can be hypothesized to maintain genetic diversity of B.

### 6.3 Model 3: removal of damage

In model 3, free A can generate a new B subunit. Here, B sustains damage (B*) which reduces the activity of the AB replicator. Instability of B leads to free A subunit, which then generates a new B. Modeling was conducted with the assumption that B and B* have the same instability. Under these conditions, both stochastic modelling and deterministic modeling using rate equations revealed that instability of B and B* is favorable if the activity of the damaged AB* replicator is close to zero (Li et al., 2018; Qu et al., 2021). In this way, the basal turnover rate of B reduces the accumulation of damage and favors replicative fitness.

In cells, in addition to the role of basal turnover rate in reducing the accumulation of damage, certain types of damaged macromolecules, such as oxidized proteins, are targeted for more rapid degradation (Pickering and Davies, 2012). These targeted degradation mechanisms are also consistent with SAI model 3.

## 7 Classical models of autopoiesis and cellular automata suggest SAI

As discussed above, recent computer simulations of a hypothetical AB replicator, involving both stochastic models and deterministic models, revealed the beneficial effects of B instability in SAI models 1–3 (Li et al., 2018; Qu et al., 2021). Several classical methods have been used to computationally simulate cells and replicators, and these methods also revealed examples of SAI.

### 7.1 Autopoiesis modeling

**FIGURE 5:**
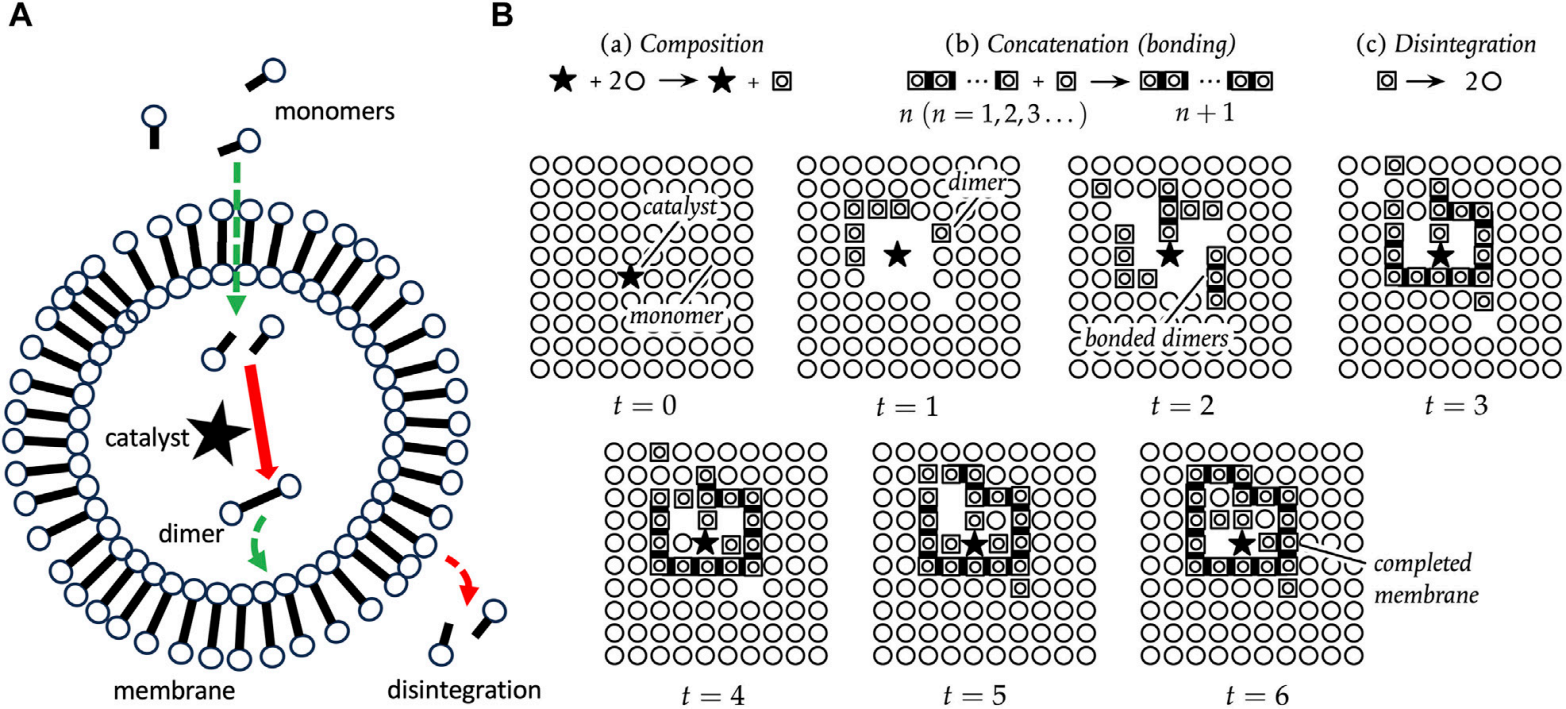
Autopoiesis. **(A)** Autopoiesis model for cellular life. The catalyst, indicated by a black star, catalyzes the dimerization of monomers into dimers, and the dimers spontaneously associate to form the membrane. The membrane dimers spontaneously degrade, and therefore continuous production of dimers is required to maintain the membrane. Monomers can diffuse through the membrane, but the catalyst cannot. **(B)** Pioneering computational simulation of autopoiesis. The black star indicates the catalyst, circles represent monomers, boxed circles represent dimers, and dark bars indicate dimers concatenated into chains. There are three processes, as indicated at the top: (a) *Composition*, in which the catalyst forms dimers; (b) *Concatenation*, the spontaneous formation of chains of dimers; and (c) *Disintegration*, the spontaneous breakdown of dimers to monomers. Additional rules include the ability of dimers to move, the ability of monomers to pass through chains, and the ability of the catalyst to move, but not pass through chains. After 6 iterations of the operations, a completed chain formed surrounding the catalyst, illustrating the emergence of a distinct autopoietic unit within the environment. The panel **(B)** is reproduced without changes from Cornish-Bowden and Cardenas (2020), who based their figure on original diagrams in Varela et al. (1974).

One classical model for the cell is called autopoiesis (Varela et al., 1974; Cornish-Bowden and Cardenas, 2020). An autopoietic system is defined as a network of synthesis and degradation processes. These processes are organized such that they continuously regenerate components, and constitute a distinct unit within the environment (Figure 5A). The essential characteristics of an autopoietic system have been described as a semipermeable boundary (or membrane), a network of reactions, and the interdependence of the boundary and the network of reactions. A pioneering computational simulation of an autopoietic system involved a two-dimensional array of monomers, a catalyst that creates dimers, spontaneous concatenation of dimers to form chains of arbitrary length, and spontaneous disintegration of chains back into monomers (Figure 5B). Additional rules included the ability of dimers to move, the ability of monomers to pass through chains, and the ability of the catalyst to move, but not pass through chains. After 6 iterations of the operations, a completed chain formed surrounding the catalyst, illustrating the emergence of a distinct autopoietic unit within the environment (Figure 5B).

Because monomers can diffuse into the autopoietic unit, the catalyst can form new dimers inside the unit. These new dimers then replace the decaying dimers of the boundary, thereby maintaining the closed boundary. Varying the decay rate for dimers was found to control the maximum size for a viable boundary structure. In this way, SAI of the dimers promotes formation of the autopoietic unit in two ways. First, SAI of the dimer destabilizes unbounded structures, including mis-formed membrane fragments. This might be interpreted in terms of model 3, removal of damage (Figure 4C). Second, SAI of the dimers facilitates the assembly of a boundary of defined size. If the dimer is too stable, the membrane may be too large, and might be unstable in the face of environmental agitation. If the dimer is too unstable, the membrane may be too small or not form at all. This modulation of boundary size might be interpreted in terms of model 2, where SAI of the dimer enables adaptation to the environment (Figure 4A).

### 7.2 Cellular automata modelling

**FIGURE 6:**
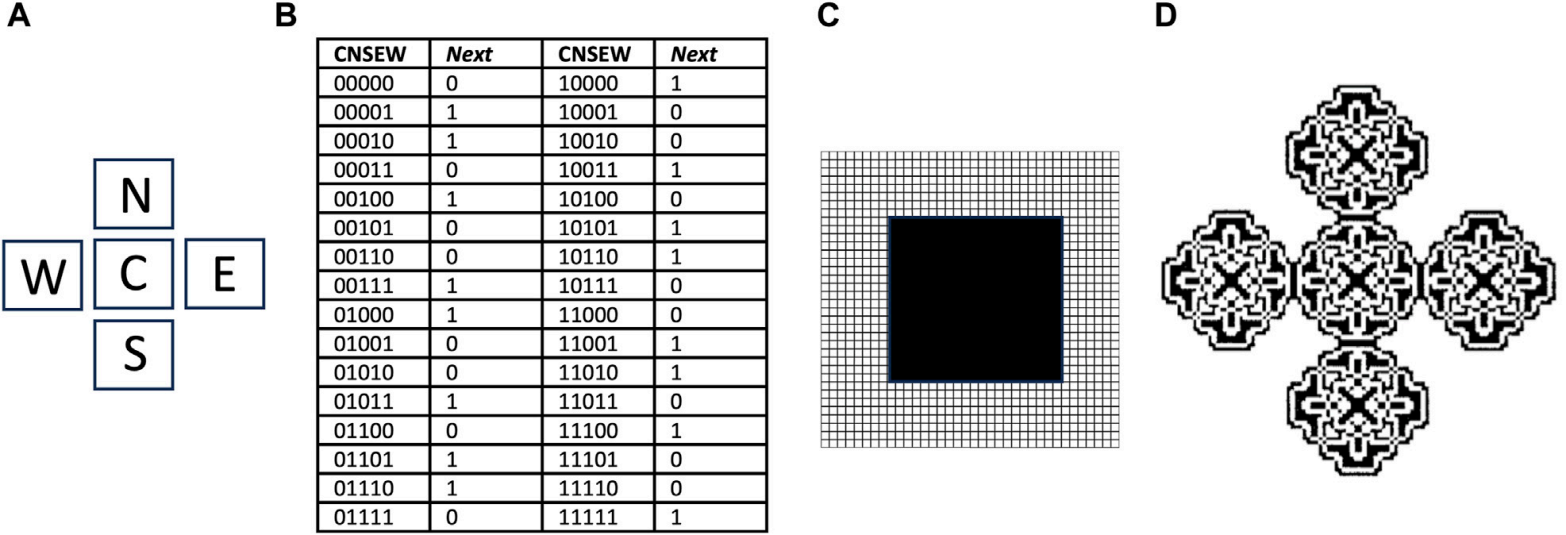
Cellular automata and the parity rule. The cellular space is two-dimensional, with a five-cell neighborhood, 32 transition rules, and two possible cell states, 0 and 1. **(A)** The five-cell neighborhood, Center (C), North (N), South (S), East (E) and West (W). **(B)** Transitions table. CNSEW is the current state of the five-cell neighborhood, and *Next* is the state of the C cell at the next time step. **(C)** Starting state for simulation. A 20 × 20 square grid of cells in state 1 (black), is surrounded by a field of cells in state 0 (white) that extends in each direction. **(D)** Resulting pattern after 90 time steps. Panel (D) is reproduced with permission from Sipper (1998).

Cellular automata were developed in the 1950s by von Neumann, as a method for computational modeling of self-replicating structures (Reggia et al., 1998; Sipper, 1998; von Neumann and Burks, 1966). Similar to the autopoiesis modeling described above, the cellular automata is an abstract logical machine that functions in a two-dimensional cellular space (Figure 6). The cells can exist in one of several possible states, and while most cells are typically inactive, the subset of cells that are active are called components. The replicator is represented by a specific configuration of contiguous active cells. At each time step of the model, the state of each cell is determined based upon its current state and the states of adjacent cells, according to a series of rules specified by the researcher. In this way, a replicator can go through a series of steps to create new copies of itself, by altering the state of adjacent cells.

For example, a relatively simple cellular automata is described by the “parity rule”, which does not involve SAI. The parity rule specifies cells with only two possible states (0 or 1), organized in a five-cell neighborhood, with the five positions named Center (C), North (N), East (E), South (S), and West (W) (Figure 6A). At each time step, the C cell is assigned state 1 if the sum of its current state plus each of its four neighbor’s states is an odd number, and is assigned state 0 if that sum is even. The table shows each of the 32 possible configurations, half of which code for state 0, and half of which code for state 1, and these are referred to as transition rules (Figure 6B). The simulation presented begins with a solid square of 20 × 20 cells, all in state 1 (black), surrounded by a field of cells in state 0 (white) (Figure 6C). After 90 time steps of these rules, the square grew into an elaborate pattern consisting of five replicated sub-structures (Figure 6D) (Sipper, 1998); however, these substructures remain connected, and do not represent distinct replicators. By beginning with a more complex starting structure, and adding additional rules, including SAI, completely self-replicating cellular automata can be generated.

### 7.3 SAI in cellular automata

**FIGURE 7:**
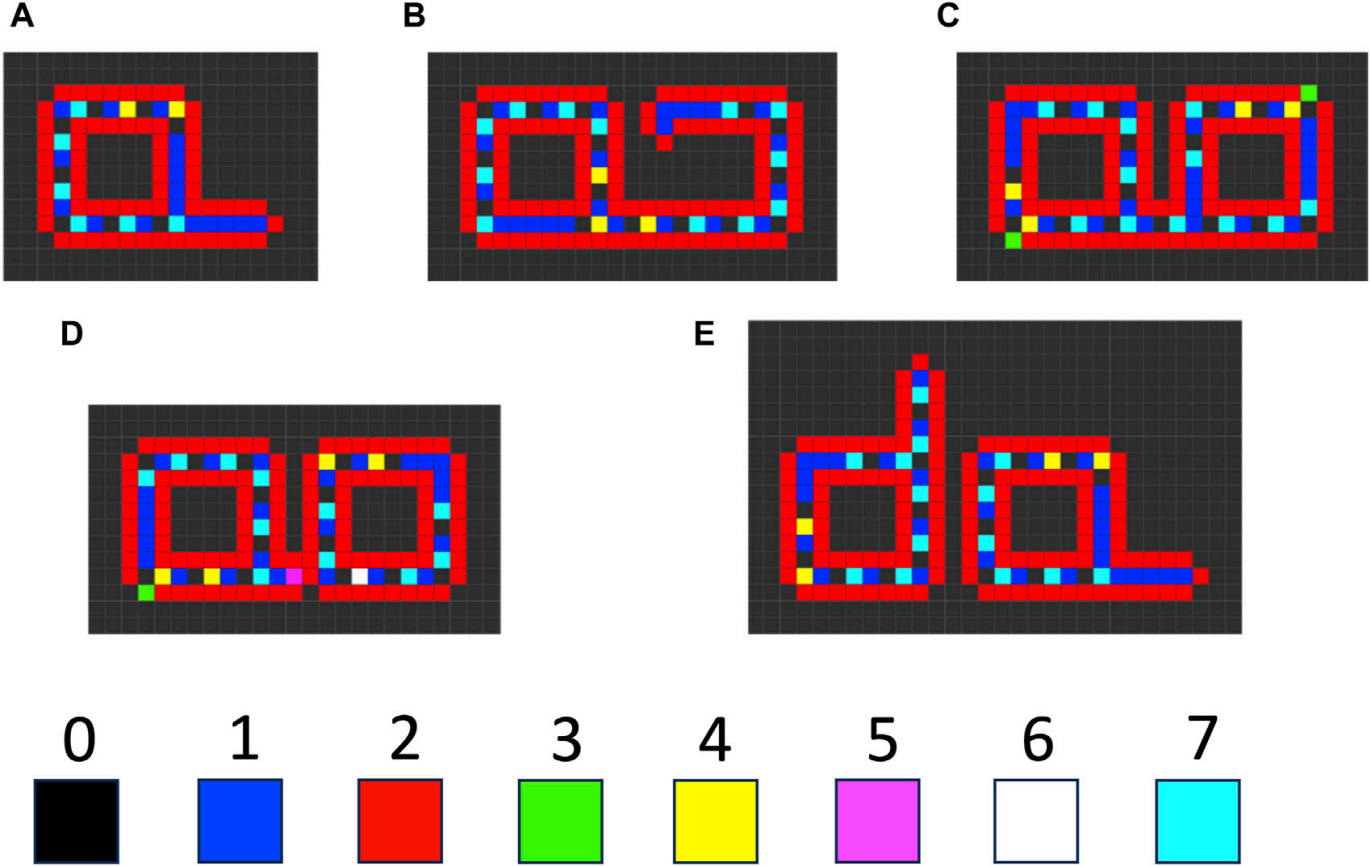
Langton’s loop cellular automata. The cellular space is two-dimensional, with a five-cell neighborhood, 219 transition rules, and 8 possible cell states (0–7). **(A)** The starting Langton loop (t = 0). **(B)** Extension of the arm and beginning of the third turn (t = 105). **(C)** Completion of the closed daughter loop (t = 124). **(D)** Beginning degradation of the connector region, associated with the appearance of cell state #5 (t = 127). **(E)** Completed degradation of the connector, and formation of two complete Langton’s loops (t = 151). The simulation was generated using Golly version 4.2.1 for the Web, ^©^ 2005–2022 The Golly Gang: Andrew Trevorrow, Tom Rokicki, Tim Hutton, Dave Greene, Jason Summers, Maks Verver, Robert Munafo, Chris Rowett. (http://golly.sourceforge.net/). Steps in the simulation correspond to those from Physica 10D, Langton, C.G, SELF-REPRODUCTION IN CELLULAR AUTOMATA, Pages 135–144, Copyright (1984), with permission from Elsevier.

There are several examples where instability of a component of a self-replicating cellular automata provides a benefit to replication, consistent with SAI. For example, Langton’s loop is a classical self-replicating cellular automata, resembling a square closed loop with a short arm (Figure 7A) (Langton, 1984). It is implemented in a 5-neighbor space, with 8 possible states for each cell, and 175 transition rules. Additional functions are encoded by the sequence of states in the core of the loop. For example, the sequence “7 0” signals for extension of the arm by 1 cell. Replication begins with the extension of the arm, which then takes three left-hand turns (Figure 7B), until it forms a second closed loop (Figure 7C). The connection between the parent loop and the daughter loop then degrades, based on a signal encoded by cell state #5 (Figure 7D). Degradation of the connection allows the two loops to separate, and each loop then re-forms the short arm to yield two complete replicators (Figure 7E). The entire process requires 151 time steps. In this way, SAI of an intermediate structure formed during replication enables separation of the progeny replicators. This may be interpreted as an example of model 2, where loss of B enables the replicator to adapt to a changing environment, and the change in environment is a step in the division process. Indeed, Langton referred to the unstable connector region as the “umbilical cord” (Langton, 1984). This mechanism is also reminiscent of the degradation of cell components at the midline during bacterial cytokinesis.

When self-replicating cellular automata such as Langton’s loop proliferate and begin to fill the two-dimensional space, competition for inactive cells can cause one automata to stall during its replication process, thereby preventing its further replication. Modification of the Langton model with additional rules and cell states yields automata that can avoid such stalling events. When the growing arm of these automata encounters an obstacle and stalls, the arm degrades, thereby allowing the automata to potentially re-start replication in another orientation, or when the obstacle has moved (Tempesti, 1995; Huang et al., 2013). In this way, SAI of the extended automata arm component favors replicative fitness. This mechanism might be interpreted in terms of model 3, removal of damage (Figure 4C), where the stalled arm represents a damaged subunit. In addition, this mechanism might also be interpreted in terms of model 2, where the degradation of arm allows the replicator to adapt to a changing environment involving moving obstacles.

## 8 SAI in cells and organisms

In cells, the majority of macromolecules are degraded and regenerated at a finite rate. The realization that molecular turnover is a fundamental feature of biology is generally attributed to Schoenheimer (Schoenheimer, 1942), who observed that “all constituents of living matter, whether functional or structural, of simple or of complex constitution, are in a steady state of rapid flux.” As discussed above, SAI has potential benefits in addition to mobilization of building blocks and the generation of energy. There are several mechanisms in cell physiology where subunit instability provides such a selective advantage, consistent with the three basic SAI models described above.

### 8.1 Short-lived transcription factors and adaptation to a changing environment

Proteins in cells vary dramatically in their rates of degradation, and their stability is encoded in the primary sequence through motifs that are recognized by protein degradation regulatory factors (Laney and Hochstrasser, 1999; Fuertes et al., 2003; Kaushik and Cuervo, 2018; Varshavsky, 2019). Many transcription factor proteins and signaling proteins have half-lives on the order of minutes, which enables a rapid activation and inactivation in response to a changing environment. For example, in eukaryotic cells, the transcription factor Nrf2 has a half-life of approximately 20 min (Kobayashi et al., 2004). Upon oxidative stress, Nrf2 degradation is inhibited, and the stabilized Nrf2 activates increased transcription and expression of components of the 20S proteasome, which enables adaptation of the cell and the organism to the increased oxidative stress (Pickering et al., 2012). Similarly, the central regulator of metabolism and stress response p53 is continually made and degraded under normal conditions (Bang et al., 2019). Upon appropriate environmental stress signals, the degradation of p53 is inhibited, and its increased activity enables adaptation to the changing environment. The regulation of Nrf2 and p53 at the level of degradation rate enables a more rapid change in protein abundance and activity in response to stress than does a change in transcription, which requires the additional steps of transcription, mRNA processing, mRNA transport, and translation. Indeed, creating highly sensitive sensors of stress appears to be a common role for SAI in cells, as discussed further below. In summary, in these examples, SAI of subunit B (where B is Nrf2 or p53), enables adaptation to a changing environment by specifying the functional state of the replicator, as in model 2 (Figure 4A).

### 8.2 Short-lived cyclin proteins and regulation of the eukaryotic cell cycle

In the cellular replicator, the degradation of specific proteins is required for cell division. For example, in eukaryotes, cyclin proteins bind and activate cyclin-dependent kinases (CDKs), which in turn regulate entry and exit from the phases of the cell cycle. In this way, regulated synthesis and degradation of the cyclin proteins is required for normal progression through the cell cycle, including correctly regulated cell division (Heim et al., 2017). Consistent with the importance of normally regulated cyclin degradation, mutations that increase the stability of cyclin D1 mRNA or protein promote cancer in mammals (Chen and Li, 2022). Regulated protein degradation is also required for cell cycle progression in prokaryotes, including *E. coli* (LaBreck et al., 2021) and *Caulobacter crescentus* (Fatima et al., 2022). Cell division occurs in response to cell growth, which in turn can be considered a response to an appropriately nutrient-rich environment. In this way, SAI of subunit B, where B is the cyclin protein (or the cyclin mRNA), enables adaptation to a changing environment by specifying the functional state of the replicator, as in model 2 (Figure 4A).

### 8.3 Consequences of non-optimal protein stability in cells and organisms

Correct regulation of the optimal protein stability is critical for normal cell and organism function. Mutations in human proteins that decrease their stability below the optimal level often lead to loss of function and disease (Waters, 2001). For example, mutations that decrease the stability of the cytosolic liver enzyme phenylalanine hydroxylase cause the disease phenylketonuria. In turn, mutations that increase protein stability above the optimal level can also disrupt normal function and lead to disease. For example, beta-catenin protein signaling promotes cell growth, and beta-catenin activity is normally modulated by proteasomal degradation. Specific mutations in the beta-catenin protein that make it refractory to degradation cause excessive growth signaling that contributes to the progression of colorectal cancer (Sparks et al., 1998; Hanna et al., 2019).

### 8.4 Removal of macromolecule damage

The degradation of protein is required for replacement of damaged molecules, as in model 3 (Figure 4C), in both bacteria and eukaryotes (Pickering and Davies, 2012; Guo and Gross, 2014; Pomatto et al., 2017; Pomatto et al., 2020). In addition to any basal rate of turnover, protein misfolding and/or other damage promotes targeted destruction of the damaged protein (Pickering and Davies, 2012; Slominska-Wojewodzka and Sandvig, 2015; Balchin et al., 2016; Sala et al., 2017). Genetic, chemical or disease-associated inhibition of protein degradation pathways results in accumulation of misfolded, damaged and aggregated proteins, contributing to cessation of cell division and to cell death (Mahmoud and Chien, 2018; Lim et al., 2023).

Membrane phospholipids are subject to oxidative damage caused by endogenous metabolic byproducts, as well as from environmental oxidative stress (Pamplona, 2008; Eckl and Bresgen, 2017). Studies in cultured mammalian cells suggest that rapid constitutive recycling of membrane phospholipids, as opposed to a targeted repair, is responsible for eliminating peroxidized phospholipids, consistent with model 3 (Giron-Calle et al., 1997; Pamplona, 2008).

RNA molecules are particularly susceptible to oxidative damage and to alkylation damage (Yan and Zaher, 2019). In *E. coli*, the majority of mRNA molecules have a basal half-life between 3 and 8 min, which is expected to prevent any significant accumulation of damaged molecules, consistent with model 3 (Bernstein et al., 2002; Deutscher, 2006). However, functional RNAs such as tRNAs and ribosomal RNAs, and several eukaryotic mRNAs are longer lived, and accumulation of damaged molecules may have negative consequences, such as ribosome stalling and production of abnormal proteins. For example, presence of the oxidative damage product 8-oxo-guanine in eukaryotic mRNAs is reported to stall translation, resulting in production of truncated proteins. Ribosome stalling activates a quality-control process called no-go decay, which targets the associated mRNA for endonucleolytic cleavage and degradation (Monaghan et al., 2023), again consistent with model 3.

### 8.5 Non-covalent assemblies

As mentioned above, disruption of static assemblies is common in cells, and might be interpreted in terms of model 2 (Figure 4A), where instability of the assembly enables the cell to adapt to the environment. For example, the ATP-dependent dissociation of HSF trimers reduces expression of hsp genes in response to the resolution of protein folding stress (Kmiecik et al., 2020). Similarly, ATP-dependent remodeling of nucleosome structures enables changes in gene transcription in response to environmental stress and other signals (Imbalzano et al., 1996; Langst and Becker, 2004; Becker and Workman, 2013).

The disruption of dynamic assemblies might also be considered an example of model 2 (Figure 4A), where instability of the structure enables the replicator to adapt to changes in the environment. For example, microtubules are long polymers formed by the self-assembly of tubulin protein dimers. Tubulin dimers bound to GTP assemble onto the tubule at the end, and the GTP is hydrolyzed to GDP shortly after incorporation. The tubule containing GDP-bound dimers is less stable and subject to depolymerization, and therefore a supply of energy in the form of GTP is required to maintain the microtubule structure (Mitchison and Kirschner, 1984; Horio and Murata, 2014). Mitchison and Kirschner hypothesized that the cell evolved GTP hydrolysis by tubulin in order to create a polymer with “built-in instability” that facilitates its function in the cell (Mitchison and Kirschner, 1984; Desai and Mitchison, 1997). The dynamic instability of the microtubule was proposed to allow the structure to grow rapidly and be stabilized by end interactions, but also shrink rapidly in response to signals such as reduced GTP concentration. This enables the dynamic changes in microtubule structure that function in processes such as cell locomotion and the movement of chromosomes during mitosis. Dynamic assemblies might therefore be interpreted as a special example of SAI. The cell as a whole can be considered a dynamic self-assembly, in that it forms through self-replication, requires a constant source of energy to maintain its structure and function, and dissipates this energy to the environment in the form of heat.

### 8.6 SAI and criticality

Criticality is a phenomenon that emerges from interactions among many elements in a group, where the group is positioned in its parameter space at the interface between two or more distinct states (Bak et al., 1987). Criticality manifests as a rapid and dramatic change in the state of the group upon appropriate perturbation. In certain situations, this can involve a rapid change from an ordered state to a chaotic state, defined as being “bounded, deterministic, nonlinear, and [having] a positive largest Lyapunov exponent, meaning that initially similar phase space trajectories diverge exponentially fast” (Toker et al., 2020). One classic example is a pile of sand, where the shape and integrity of the pile is a critical state that is maintained by interactions between the grains. Adding grains at one site in the pile can cause a local instability, which relaxes by loss of grains to an adjacent site. This can induce additional instabilities, leading to a chaotic avalanche that propagates through the pile until all sites are stable again (Tadic and Melnik, 2021). Common features of criticalities are proposed to include scale invariance, power-law behavior and fractal geometry (Marković and Gros, 2014; Tadic and Melnik, 2021).

Biological components that undergo self-assembly can exhibit criticality, often called self-organized criticality. For example, a flock of starlings may be considered a dynamic assembly, in that it self-assembles, requires a constant source of energy from previously ingested food, and dissipates this energy to the environment in the form of heat. The coordinated movement of the flock arises from local interactions between the individuals, and the propagation of this information throughout the group (Mora and Bialek, 2011; Bialek et al., 2012). The flock maintains cohesiveness and changes its movement rapidly in response to external perturbations, such as an approaching predator. Analysis of flocks of different sizes reveals that long-range correlations in changes in flight direction show scale invariance. This combination of order and high susceptibility to a change in state has been suggested to indicate criticality. Another example of self-organized criticality is microtubules. As discussed above, microtubules are a dynamic assembly that self-assembles through polymerization of tubulin dimers, and these microtubules can rapidly alternate between a state of polymerization and a state of de-polymerization (Mitchison and Kirschner, 1984). The microtubule growth dynamics display a scale invariance, where the distribution function is described by the power law D_*s*_ = A*s* ^t^, where D_*s*_ is the relative number of microtubules of length *s*, and A and t are constants (Babinec and Babincová, 1995). Therefore, microtubule dynamics exhibit scale invariance and power-law behavior consistent with criticality.

There are examples of SAI by degradation that also share features with criticality. For example, as mentioned above, SAI of the key transcription and signaling factor p53 facilitates rapid transitions between different cell signaling and metabolic states (Bang et al., 2019), consistent with model 2. p53 regulates numerous other transcription and signaling factors, and functions as a central hub in a large regulatory network (Boutelle and Attardi, 2021). Modeling of the p53 regulatory network reveals a topology that follows a power-law distribution and is scale-free (Dartnell et al., 2005; Potapov et al., 2005; Nafis et al., 2015). The regulatory network is relatively robust to altered activity of random single components, but rapidly changes state in response to altered activity of p53. In this way the p53 regulatory network may represent a criticality. In addition to the key role of p53 degradation, the p53 regulatory network involves various mechanisms for interaction between nodes, including post-translational modifications, regulation of transcription, and regulation of degradation.

The *Drosophila* gap gene regulatory network also displays features consistent with criticality (Krotov et al., 2014; Verd et al., 2019). The gap genes encode transcription factors that are mutually repressive, and they regulate the anterior-posterior patterning of segments in the early embryo. Fluctuations in gap gene expression are correlated along all or part of the length of the embryo, and these fluctuations have a non-Gaussian distribution. In addition, the pattern is reported to scale in proportion to the size of the embryo (Jaeger, 2011). The concentration of each gap protein depends on the rate of synthesis and the rate of degradation, consistent with the idea that SAI is important in generating the criticality. Finally, Vidiella et al. engineered a self-organizing criticality in *E. coli* using two transgenes encoding cross-regulatory transcription factors that compete for degradation by the ClpXP protease (Vidiella et al., 2021). In addition to enabling rapid and sensitive responses to the environment, gene regulatory networks at criticality are predicted to favor evolvability (Torres-Sosa et al., 2012; Villani et al., 2018).

Taken together, the data indicate that SAI is part of the mechanism that generates several forms of criticality, consistent with model 2, change in state.

### 8.7 Turing patterns

Reaction-diffusion mechanisms are implicated in the generation of many patterns in nature, including features of animal body plans such as the digits of the hands and feet (Gierer and Meinhardt, 1972; Raspopovic et al., 2014). In Turing’s original reaction-diffusion model (Turing, 1990), an activator P causes the production of more P, as well as the production of inhibitor S. In turn, S inhibits the production of P. If P diffuses more slowly than S, this will produce waves of concentration differences for P, thereby yielding a pattern. In the context of developmental patterning, P and S are proteins or other signaling molecules whose abundance is a function of both synthesis and degradation rates (Gierer and Meinhardt, 1972; Raspopovic et al., 2014). In this way, SAI functions as a component of many reaction-diffusion mechanisms and is important in specifying the resultant pattern, again consistent with model 2, change of state.

### 8.8 Cellular consciousness and nanobrains

It has been suggested that cells “experience valence-marked, subjective, internal, representational states” that are determinative of their actions, and that “these forms of subjective awareness, either of environmental events or of organismal internal states” represent a form of cellular “consciousness” (Baluska and Reber, 2019). The key components are thought to include excitable membranes, cytoskeletal polymers, and structurally flexible proteins. For example, microtubules have been reported to exhibit electrical conductivity that is a function of their degree of polymerization and their past history of electrical stimulation, and to therefore act as memristors (Tuszynski, 2019; Tuszynski et al., 2020; Baluska et al., 2021). The protein network that comprises the ribosome transmits long-range signals from one site in the ribosome to another during the steps of translation, and these signals are thought to include protein conformational changes and charge transfers (Timsit and Gregoire, 2021). Subcellular structures, such as the ribosome and microtubules, that integrate multiple current and past inputs have been referred to as “nanobrains” (Baluska et al., 2021; Timsit and Gregoire, 2021). Regulated degradation (SAI) of components of subcellular signaling complexes is another mechanism the cell uses to create a record of past external and internal stimuli. Examples include the stabilization of p53 and Nrf2 in response to environmental stress, as discussed above, as well as the caspase-mediated proteolysis of several nuclear pore complex subunits during cell differentiation and in response to ER stress (Cho and Hetzer, 2023). These examples suggest that SAI is one mechanism that contributes to cell “consciousness,” consistent with model 2, change of state.

## 9 SAI and minimal cells

Minimal cells retain only the most essential genes and pathways, thereby facilitating the study of basic mechanisms of biology, including SAI.

### 9.1 Mycoplasmas

Because of their parasitic mode of life, the mycoplasmas have evolved greatly reduced genomes, making them ideal models for minimal cells. For example, *M. pneumoniae* M129 is estimated to have −732 genes, including only two essential ATP-dependent proteases, the cytoplasmic Lon protease and the membrane-targeted FtsH protease (Himmelreich et al., 1996; Glass et al., 2017). Combined proteomics and RNA expression analysis of *M*. *pneumoniae* M129 after conditional depletion of FtsH and/or Lon identified 34 FtsH substrates and 62 Lon substrates (Burgos et al., 2020). Lon substrates were enriched for short-lived proteins and cell cycle functions, and Lon was found to recognize accessible hydrophobic degrons. The data suggest that Lon regulates the turnover of functional native proteins to control adaptation to a changing environment, including cell cycle phases, as in model 2 (Figure 4A). In addition, Lon-depleted cells had increased abundance of aggregated and insoluble proteins, under both control and heat-stress conditions, revealing a role for Lon in protein quality control, as in model 3 (Figure 4C). In contrast, FtsH substrates were enriched for membrane-associated proteins, and FtsH-depleted cells had compromised membrane integrity, indicating an important role for FtsH in membrane protein quality control (Burgos et al., 2020), again consistent with model 3.

In bacteria, the conserved proteins FtsZ and FtsA dynamically assemble at the cell membrane at mid-cell location in a complex called the Z-ring, and regulate cytokinesis during cell division (Adams and Errington, 2009). For example, during the cell cycle of *C. crescentus*, synthesis and degradation of these proteins is regulated such that FtsZ peaks at middle S phase, and FtsA peaks at the S/G2 transition, reminiscent of eukaryotic cyclins (Fatima et al., 2022). In *M*. *pneumoniae* M129, both FtsZ and FtsA were found to be substrates of Lon, suggesting that Lon regulates Z-ring formation and disassembly and cytokinesis in this minimal cell (Burgos et al., 2020). This requirement for protein degradation in cell replication is consistent with model 2 (Figure 4A), and is reminiscent of the requirement for degradation of the linker region during replication of the Langton loop cellular automata (Figure 7).

### 9.2 Synthetic minimal cells

Starting with the genome of *Mycoplasma mycoides* as a guide, chemical synthesis and testing of minimal viable genomes has resulted in the generation of cells with <500 genes (Hutchison et al., 2016). For example, the minimal cell JCVI-Syn3A contains 493 genes, and these include the two ATP-dependent proteases, Lon and FtsH, as well as an oligoribonuclease, consistent with the idea that protein and RNA degradation is essential for life (Glass et al., 2017; Breuer et al., 2019; Pelletier et al., 2022). Modeling of JCVI-Syn3A metabolism, based in part on analyses of the related organism *M. pneumoniae*, suggests that the entire proteome is subject to turnover (Maier et al., 2011; Breuer et al., 2019). The average protein half-life was estimated to be 25 h, with an energetic cost of 225 ATP for each protein degraded. One likely function for this proteome turnover is to eliminate damaged molecules, as in model 3 (Figure 4C). Notably, JCVI-Syn3A retains the genes encoding FtsZ and FtsA, suggesting that JCVI-Syn3A may use regulated proteolysis of FtsZ/FtsA and/or other cell cycle proteins to regulate movement through the phases of the cell cycle, as in model 2 (Figure 4A).

The half-life of mRNA in JCVI-Syn3A was estimated to be approximately 1 min, with no energetic cost for degradation (Breuer et al., 2019). This mRNA degradation may function to eliminate damaged molecules, as in model 3 (Figure 4C). Coupled with regulated transcription, mRNA degradation might also function to enable adaptation to a changing environment, including cell cycle phases, as in model 2 (Figure 4A).

## 10 Synthetic molecular replicators and pre-biotic systems

Synthetic systems have now been developed that exhibit several defining characteristics of life, including self-replication, variation and selection. For several of these systems, instability of one or more components has been found to play a beneficial role, consistent with SAI. Here, two examples are discussed.

### 10.1 Lipid micelles and vesicles

The cell is surrounded by a semi-permeable lipid bilayer, and it is hypothesized that a surrounding lipid membrane may be essential for life. There has been significant progress in the development of lipid micelles and lipid vesicles as synthetic pre-biotic systems and proto-cells (Kahana and Lancet, 2021). In several studies, a role for covalent degradation consistent with SAI has been reported.

Early studies showed that co-polymers composed of a hydrophobic moiety and a hydrophilic moiety can self-assemble into micelles in aqueous solution (Alexandridis et al., 1994). Engineering degradable moieties resulted in progressive alterations in micelle size and shape with time. For example, degradation of the hydrophobic moiety destabilized the micelles thereby decreasing their size, whereas degradation of the hydrophilic moiety produced larger micelles (Muratov and Baulin, 2012). In this way, degradation increased the complexity of the system by creating a greater variety of structures, consistent with SAI.

**FIGURE 8:**
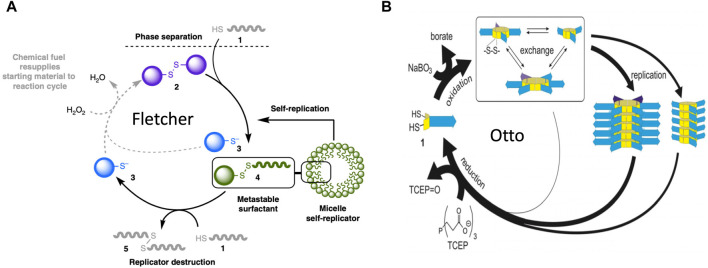
Molecular replicators. **(A)** Fletcher system replicator. In this system, reactants #1 and #2 are phase-separated, and undergo a slow thiol-disulfide exchange reaction to yield the #4 subunit (a metastable surfactant), and the #3 waste product. The #4 subunit spontaneously forms micelles. The micelles increase mixing between the phases, therefore catalyzing the formation of more micelles (self-replication). The #4 subunit undergoes degradation by a second thiol-disulfide exchange reaction, to form waste products #5 and #3. Addition of oxidizing reagent H_2_O_2_ as a “fuel” re-generates #2 from waste product #3. In this way, replication continues in an out-of-equilibrium state that consumes H_2_O_2_ fuel and substrate #1. The figure is reproduced without changes from Morrow et al., 2019 Nature Communications 10:1011. (https://creativecommons.org/licenses/by/4.0/#). **(B)** Otto system replicator. In this system, dithiol substrates #1 are oxidized by a NaBO_3_ “fuel” to produce cyclic disulfides. These disulfides undergo exchange reactions to produce rings of variable size. These cyclic-disulfide subunits then self-assemble into fibers, where the fibers differ based on the ring size of the subunits. The fibers undergo fragmentation due to mechanical energy (agitation), and the fragments seed the formation of new fibers. In this way, the fibers catalyze their own self-replication. Addition of a TCEP reducing agent causes degradation of subunits back to dithiol substrates, which can then undergo oxidation to support the formation of new subunits and new fiber replicators. Therefore, in the presence of the chemical “fuel”, replication continues in an out-of-equilibrium state. The figure is reproduced without changes from Yang et al., 2021 Angew Chem Int Ed Engl 60(20):11,344–11349 (https://creativecommons.org/licenses/by-nc-nd/4.0/deed.en#).

More recently, catalytic lipid micelles have been developed that undergo growth and self-replication (Post and Fletcher, 2020; Ameta et al., 2021; Kahana and Lancet, 2021). For example, Fletcher and coworkers developed a self-replicating micelle composed of unstable surfactant subunits, where one of the breakdown products of the subunits is combined with substrate to create new subunits (Figure 8A)(Colomer et al., 2018; Morrow et al., 2019). In this system, reactants #1 and #2 are phase-separated, and undergo a slow thiol-disulfide exchange reaction to yield the #4 subunit (a metastable surfactant), and the #3 waste product. The #4 subunit spontaneously forms micelles. The micelles increase mixing between the phases, therefore catalyzing the formation of more micelles (self-replication). The #4 subunit undergoes degradation through an energetically-favorable second thiol-disulfide exchange reaction, to form waste products #5 and #3. Addition of oxidizing reagent H_2_O_2_ as a “fuel” re-generates #2 from waste product #3. In this way, replication continues in an out-of-equilibrium state that consumes H_2_O_2_ fuel and substrate #1 (Morrow et al., 2019), and the replicator can be considered an example of a dynamic assembly. The degradation of replicator subunit #4 shows some similarity to the mobilization of building blocks through catabolic metabolism type 2 (CM2), except that energy is not required. In addition, the abundance and size of the replicator micelles at steady-state is a function of the fuel supply. In this way, the SAI of the replicator subunits facilitates adaptation to a changing environment, corresponding to model 2 (Figure 4A). SAI of the replicator micelle subunits appears similar to the autopoiesis model discussed above, where SAI of membrane subunits modulates the size of the membrane.

### 10.2 Self-assembling fibers

Otto and coworkers developed a system where dithiol substrates are oxidized by a NaBO_3_ “fuel” to produce cyclic disulfides of variable ring size (Yang et al., 2021) (Figure 8B). These cyclic-disulfide subunits then self-assemble into fibers, where the fibers differ based on the ring size of the subunits. The fibers undergo fragmentation due to mechanical energy (agitation), and the fragments seed the formation of new fibers. In this way, the fibers catalyze their own self-replication. Addition of a TCEP reducing agent causes degradation of subunits back to dithiol substrates, which can then undergo oxidation to support the formation of new subunits and new fiber replicators. Therefore, in the presence of the chemical “fuel”, replication continues in an out-of-equilibrium state. Remarkably, as the system cycles it becomes more complex, in that the replicator with the greater molecular complexity (greater subunit ring size) becomes increasingly abundant, even though it replicates slower than its smaller competitor. This is because the smaller replicator is more unstable than the larger replicator (Yang et al., 2021). Therefore, instability of the replicator subunits increases complexity of the system, generally consistent with SAI. The degradation and re-cycling of the replicator monomers might be considered an example of mobilizing building blocks through catabolic metabolism type 2 (CM2). In addition, the ability of a breakdown product from one replicator lineage to favor the replication of another replicator lineage shows some similarity to model 1 (Figure 3).

## 11 SAI promotes maintenance of genetic diversity

There are several mechanisms which indicate that SAI of a cell component can promote the maintenance of genetic diversity, including mechanisms in which SAI creates asymmetric inheritance.

### 11.1 Toxin/antitoxin systems and gene maintenance

**FIGURE 9:**
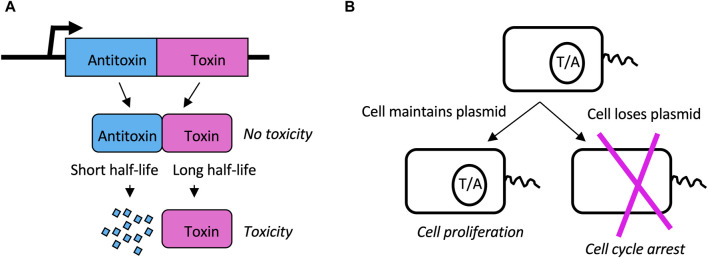
Toxin/Antitoxin (TA) systems. **(A)** TA gene pairs. The TA gene pair is co-transcribed to produce antitoxin and toxin, respectively. Antitoxin binds to and inhibits the toxin, thereby preventing toxicity. However, the toxin has a long half-life while the antitoxin has a short half-life. Therefore, without a sufficient supply of new antitoxin, the toxin will cause cell cycle arrest and possible cell death. **(B)** Plasmid addiction. TA pairs can reside on bacterial plasmids. When the cell divides, both daughters inherit both toxin and antitoxin present in the mother cell. However, because antitoxin has a shorter half-life than toxin, if the cell does not inherit the plasmid to express new antitoxin, the cell will undergo cell cycle arrest and possible cell death. In this way the TA gene pair favors its own maintenance as well as the maintenance of the plasmid (or chromosome) on which it resides.

Toxin/antitoxin (TA) systems consist of a relatively stable toxin and a short-lived antitoxin (Sonika et al., 2023). The toxin and antitoxin are encoded as a tightly-linked gene-pair, such as in a single bacterial operon. During cell division, if a daughter cell inherits the gene pair, that cell continues to express antitoxin, and continues normal metabolism and cell division. However, if a daughter cell should fail to inherit the gene pair, it is unable to express new antitoxin, and the existing toxin causes cell cycle arrest and potentially cell death (Figure 9) (Song and Wood, 2018). In this way, the TA gene-pair favors its own maintenance, as well as the maintenance of any genes to which it might be linked. Since their discovery on bacterial plasmids, TA gene pairs have been identified in the genomes of all sequenced bacteria, in numbers ranging from four to over 40, with the exception of obligate intracellular pathogens that appear to have shed these genes. At least 8 different types of TA systems have been described, based on the mechanism of toxicity, the organization of the gene pair, and whether the toxin and/or antitoxin functions as a protein or as an RNA (Sonika et al., 2023).

In addition to favoring their own maintenance, the TA pairs are also implicated in benefiting the bacterial host in several ways, including phage resistance, stress response, and pathogenicity (Kelly et al., 2023; Pan et al., 2023). For example, the *hok* (toxin) and *sok* (antitoxin) TA pair are found on the R1 plasmid in *E. coli* (Gerdes et al., 1986). In addition to stabilizing the maintenance of the R1 plasmid, the *hok/sok* system also inhibits replication of the T4 bacteriophage (Wilmaerts et al., 2018; Kelly et al., 2023). T4 phage infection causes reduced global transcription in the host cell, and reduced transcription of the *hok/sok* locus causes increased relative abundance of the toxin. The toxin then disrupts the cell membrane, causing a loss of proton motive force, and inhibition of phage replication. Therefore, TA pairs represent another example of where SAI creates a highly sensitive sensor for stress. Notably, the minimal cell JCVI-Syn3B contains 473 genes, with no detectable homology to known TA pairs, and therefore TA pairs do not appear to be essential for life (Hutchison et al., 2016; Hossain et al., 2021).

The beneficial effects of TA pairs for phage resistance, stress response, and pathogenicity might be interpreted in terms of SAI model 2, where instability of a subunit favors the cells response to the environment (Figure 4A). In addition, this mechanism might be generally interpreted in terms of model 2 maintenance of genetic diversity (Figure 4B), in that instability of B selects for maintenance of the TA gene pair and any linked genes.

### 11.2 SAI enables asymmetric inheritance mechanisms

**FIGURE 10:**
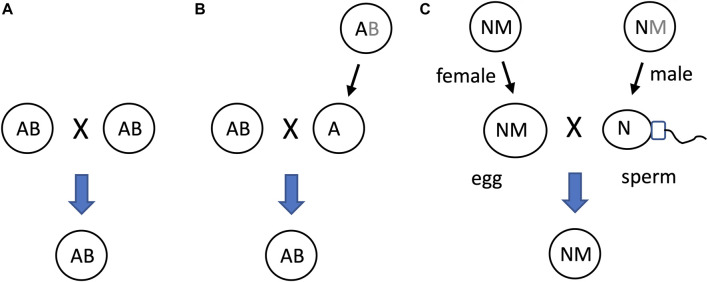
SAI of the mitochondria facilitates asymmetric inheritance. In cells that combine their genetic information to create progeny, SAI can create asymmetric inheritance. **(A)** If both cells contain both A and B, then both cells can contribute A and B to the progeny. **(B)** Instability of B creates a parent that contains only A. Therefore, this cell can contribute only A to the progeny, thereby yielding asymmetric inheritance of B. **(C)** Instability of the mitochondria (M) in the male germline creates sperm containing N, with little or no heritable M; what little paternal M that reaches the fertilized egg is destroyed, thereby yielding asymmetric inheritance of M.

Another potential significance of SAI rule is that it can facilitate beneficial asymmetric inheritance mechanisms. For example, the nucleus and the mitochondria collaborate as subunits of the eukaryotic cellular replicator (Rand, 2011; Pickles et al., 2018; Klucnika and Ma, 2019). In eukaryotes that reproduce sexually, including humans and *Drosophila*, mitochondria are typically asymmetrically inherited, in that they are transmitted to offspring through the egg, with little to no transmission through the sperm. In eukaryotic cells, the mitochondria are continually degraded and re-generated, whereas the nucleus is typically stable (Pickles et al., 2018). The shorter half-life of the mitochondria (subunit B in this example) relative to the nucleus (subunit A in this example) facilitates the creation of sperm and zygote containing nucleus, but few, if any, paternal mitochondria (Figure 10).

The mechanisms for instability of the paternal mitochondria involve degradation during spermatogenesis and/or in the fertilized egg. For example, in *Drosophila*, most mitochondria and mitochondrial genomes are eliminated from the developing spermatid in an apoptotic-like process during spermatid individualization (Fabrizio et al., 1998; Arama et al., 2003; Arama et al., 2006; Yu et al., 2017). Ectopic expression of the baculovirus caspase-inhibitor gene p35, or reduced expression of the pro-apoptotic genes cytochrome C, Apaf, Caspase-9 or mitochondrial endonuclease G disrupted this process (Yu et al., 2017). The few remaining paternal mitochondria that enter the egg are destroyed by the maternal autophagy pathway (Politi et al., 2014). In humans, the great majority of mitochondrial DNA is also eliminated during the formation of the sperm (Lee et al., 2023). In *C. elegans*, the conserved apoptosis regulatory factor mitochondrial endonuclease G acts in the fertilized egg to promote degradation of the paternal mitochondria by the maternal autophagy and proteosomal degradation pathways (Zhou et al., 2016; Lim et al., 2019). Similarly, paternal mitochondria are reported to be eliminated by the autophagy pathway in early mouse embryos (Rojansky et al., 2016). In summary, conserved apoptotic regulatory factors and the autophagy pathway mediate SAI of the paternal mitochondria across multiple species.

The asymmetric inheritance of the mitochondria has been proposed by our group (Tower, 2006; Tower, 2015) and others (Hadjivasiliou et al., 2012; Havird et al., 2015; Radzvilavicius et al., 2016) to promote genetic diversity and evolution, including the evolution of sex.

### 11.3 SAI and asymmetric mitochondrial inheritance can promote genetic diversity

**FIGURE 11:**
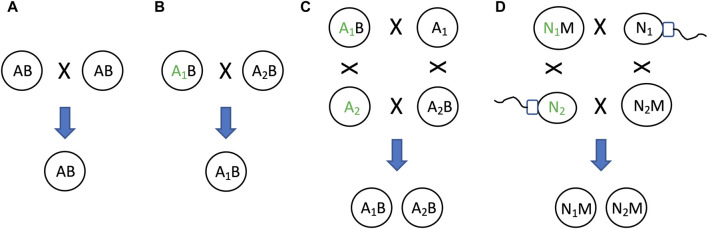
SAI promotes genetic diversity. In cells that combine their genetic information to create progeny, SAI can promote the maintenance of genetic diversity. **(A)** If both parent cells contain A and B, both cells can contribute A and B to offspring. **(B)** Selection for allele of A. If the two parent cells contain different alleles of A (A_1_ and A_2_, respectively), and A_1_ confers an advantage for reproductive fitness (indicated by green font), then A_1_ will be preferentially transmitted to offspring. **(C)** If B is unstable, this creates two distinct states for A, one state with B and one state without B. If A_1_ confers an advantage for reproductive fitness in the presence of B, and A_2_ confers an advantage for reproductive fitness in the absence of B, then both A_1_ and A_2_ will be preferentially transmitted to offspring, thereby maintaining genetic diversity of A in the population. **(D)** Egg and sperm. Because mitochondria (M) is unstable, the nucleus (N) can exist in two distinct states, one state with M (egg), and one state without M (sperm). There are two alleles for N, N_1_ and N_2_. If N_1_ confers an advantage for reproductive fitness in the presence of M, and N_2_ confers an advantage for reproductive fitness in the absence of M, then both N_1_ and N_2_ will be preferentially transmitted to offspring, thereby maintaining genetic diversity of N in the population.

As discussed above, the relative instability of the mitochondria (B in this example) relative to the nucleus (A in this example), creates two states: one state with heritable mitochondria (the AB state, representing the egg), and one state without heritable mitochondria (the A alone state, representing the sperm)(Figure 10C). This situation can be interpreted in terms of model 2, as a mechanism that maintains genetic diversity of A (Figure 4B). Because the two states, AB and A alone, can respond differently to selection on A, this can maintain genetic diversity of A in the population (Figure 11). For example, if allele A_1_ is selectively advantageous in the presence of B, and allele A_2_ is selectively advantageous in the absence of B, then SAI of B can promote maintenance of both alleles of A in the population (Figures 11C,D). The potential consequences of uniparental mitochondrial inheritance for the maintenance of genetic diversity has engendered significant interest and research.

Because mitochondria are transmitted almost exclusively from the mother, natural selection can only act to optimize mitochondrial gene function and nuclear/mitochondrial gene interactions for the female. The male therefore inherits a mitochondrial genome that is not optimized for function in the male (the Frank and Hurst hypothesis, sometimes called “mother’s curse”) (Frank and Hurst, 1996; Gemmell et al., 2004). Natural selection is expected to act on nuclear genes in the male to select for alleles that can compensate for the defective mitochondria (Clark, 1984; Babcock and Asmussen, 1996; Babcock and Asmussen, 1998; Rand et al., 2001; Tower, 2006). In turn, in the next-generation, these male-selected alleles are likely to be non-optimal in the female (Tower, 2006). Fishers principle describes a mechanism that limits the extent to which male-harming (or female-harming) alleles can accumulate and distort the typical 1:1 sex ratio (Carvalho et al., 1998; Uller et al., 2007). For example, if females become more abundant, males will have access to more mates, thereby increasing male fitness. This will create selection for parents that produce more male progeny, thereby selecting against male-harming alleles, and/or selecting for compensatory alleles. This system is hypothesized to maintain segregating alleles (genetic diversity) that promote sexual differentiation and evolution, as well as promoting aging and aging-associated disease (Tower, 2006; Havird et al., 2015; Tower, 2015; Havird et al., 2019; Tower et al., 2020).

Consistent with these models, several mitochondrial-regulatory gene mutations with sex-biased, and sometimes sexually-opposite effects on stress resistance and life span have been identified (Garcia et al., 2017; Mank, 2017; Ventura-Clapier et al., 2017; Rusuwa et al., 2022). These include p53 in *Drosophila* (Waskar et al., 2009) and humans (Rubin et al., 2020), and Foxo/Daf-16, which appears to act preferentially in males of both *Drosophila* and *C. elegans* (Gems and Riddle, 2000; Shen and Tower, 2010; Ancell and Pires-daSilva, 2017). Nuclear/mitochondrial gene allele linkage disequilibrium is reported to exist throughout the human genome (Sloan et al., 2015). Consistent with this conclusion, Mehrpour et al. identified several nuclear genes with alleles that display departure from expected Hardy-Weinberg ratios depending upon the mitochondria genotype (Mehrpour et al., 2020).

Finally, the shorter half-life of mitochondria relative to nucleus is also consistent with the non-exclusive model 3, for elimination of damaged mitochondria (Figure 4C). Damaged and non-functional mitochondria are targeted for destruction by a specialized form of macroautophagy called mitophagy (Tower, 2015; Picca et al., 2023). The damaged mitochondria are first enveloped by a surrounding membrane to form the autophagosome, and the autophagosome then fuses with the lysosome to mediate degradation of the mitochondrial material.

### 11.4 SAI and the evolution of the eukaryotic cell through endosymbiosis

**FIGURE 12:**
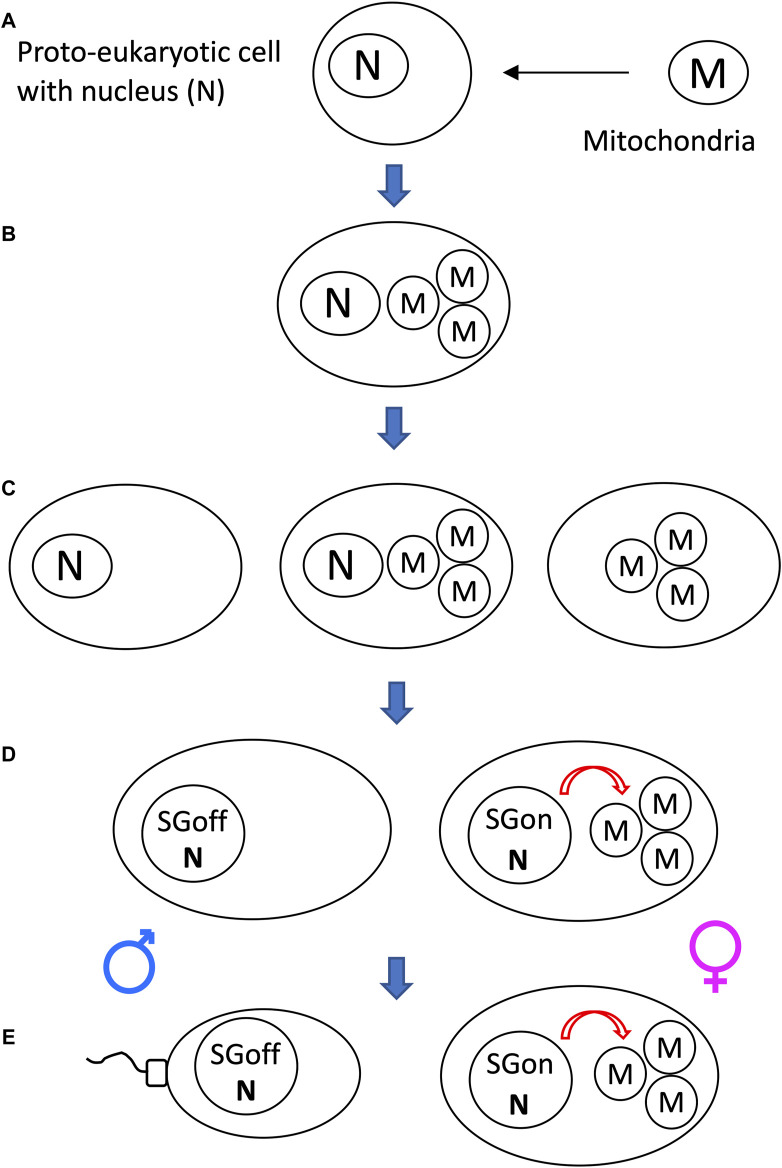
SAI and the evolution of the eukaryotic cell through endosymbiosis. **(A)** The mitochondria first invades the proto-eukaryotic cell. **(B)** Presence of nucleus and mitochondria creates the opportunity for interactions that might benefit the reproductive fitness of both. **(C)** Presence of nucleus and mitochondria also creates potential competition for resources and for transmission to daughter cells. SAI of the mitochondria can be interpreted as a mechanism that resolves this conflict, thereby enabling the nucleus and mitochondria to evolve as separate entities and collaborate in a mutually beneficial symbiosis. **(D)** SAI of the mitochondria relative to the nucleus makes the mitochondria dependent upon the nucleus for continued maintenance, thereby selecting for a switch gene (SG) in the nucleus that regulates mitochondrial maintenance. **(E)** SAI of the mitochondria facilitates asymmetric inheritance (as in Figure 10C), thereby creating the male and female state, and selecting for the SG on/off state to control mitochondrial transmission and determine the sexes.

As discussed above, SAI of the mitochondria is hypothesized to enable asymmetric inheritance, promote the maintenance of genetic diversity, and to drive evolution, including the evolution of the sexes. Consistent with these ideas, SAI of the mitochondria is hypothesized to have been a critical step in the origin of the eukaryotic cell (Tower, 2006). When the mitochondria first invaded the proto-eukaryotic cell (Figure 12A), this created the opportunity for mutually beneficial interactions between nucleus and mitochondria that might benefit the reproductive fitness of both (Figure 12B) (Gray, 2017). For example, the increased internal membrane area contributed by the mitochondria is hypothesized to have enabled greater energy production by the cell per gene (Lane, 2020). At the same time, this situation created potential competition between the nucleus and mitochondria for resources and for transmission to daughter cells (Figure 12C). SAI of the mitochondria can be interpreted as a mechanism that resolves this conflict, thereby enabling the nucleus and mitochondria to evolve as separate entities and collaborate in a mutually beneficial symbiosis. In this hypothesis, SAI of the mitochondria relative to the nucleus makes the mitochondria dependent upon the nucleus for continued maintenance, thereby selecting for a switch gene (SG) in the nucleus that regulates mitochondrial maintenance (Figure 12D). At the same time, SAI of the mitochondria facilitates asymmetric inheritance (as in Figure 9C), thereby creating the male and female state, and selecting for the SG on/off state to control mitochondrial transmission and determine the sexes (Figure 12E) (Tower, 2017). Because SAI of the mitochondria creates two distinct states that are subject to different selective pressures, i.e., female with heritable mitochondria, and male lacking heritable mitochondria, SAI of the mitochondria promotes maintenance of genetic diversity and drives evolution, including the evolution of the sexes, as described above. This corresponds to model 2, where instability of the subunit favors maintenance of genetic diversity (Figure 4B).

## 12 SAI at other levels of biological organization

There are additional mechanisms in biology that involve unstable and/or asymmetrically inherited components, that might also represent examples of SAI. A subset of these possible examples are described here.

### 12.1 Telomere erosion

Telomeres are specialized structures located at the end of linear chromosomes in eukaryotic cells, and function to protect the chromosome ends from maladaptive ligation events (Chakravarti et al., 2021). Extensive research reveals that telomeres are complex organelles that function to sense various forms of stress, and to orchestrate cellular stress responses (Bettin et al., 2019; Victorelli and Passos, 2020; Jacome Burbano and Gilson, 2021; Eppard et al., 2023). Telomeres are typically composed of multiple repeats of short G-rich sequences, and because of the DNA end-replication problem, the telomeres become shorter with each cell division in somatic cells. In humans and many multicellular eukaryotes, extensive telomere erosion association with abnormally frequent cell division causes the cell to enter either irreversible cell cycle arrest (replicative senescence), or programmed cell death (PCD), thereby protecting the organism from cancer progression. These observations suggest that telomere erosion may be an example of SAI, where the benefit is observed at the level of the multicellular organism. In addition, the telomeric sequences are also sensitive to other types of DNA damaging stress, including moderate oxidative stress, and this can signal increased DNA repair and/or downregulated mitochondrial metabolism to favor cell survival. These mechanisms suggest that that telomere erosion may also be an example of SAI at the level of the individual cell, and represents another example of where SAI creates a highly sensitive sensor for internal stress. In summary, telomere erosion may be considered an example of model 2, where instability of the subunit enables adaptation to the environment (Figure 4A).

### 12.2 PCD and apoptosis

In humans and many other multicellular eukaryotes, PCD plays a critical role in resistance to cancer, as discussed above. In addition, PCD is required for the normal turnover of old and damaged cells in several tissues, including the intestinal epithelium, blood, epidermis, lungs and kidney (Elmore, 2007; Gunther et al., 2013; Kile, 2014). If cells are considered as subunits of the multicellular replicator, then the removal of damaged cells might be considered an example of model 3 (Figure 4C).

PCD also plays important roles in the formation of structures in humans and other multicellular organisms (Zakeri et al., 2015). For example, during metamorphosis of the frog, the pollywog tail is degraded through PCD (Nakajima et al., 2005; Ishizuya-Oka, 2011). The loss of the tail favors function of the animal in its new environment, corresponding to model 2 (Figure 4A). In humans, PCD of cells in the developing hands and feet creates the spaces between the digits (Lorda-Diez et al., 2015). The cells that originally surround the developing digits are thought to help regulate digit development through cell-cell signaling and physical interactions, and are particularly important in determining correct spacing of the digits. These processes may also correspond to model 2 (Figure 4A), where the subunit is beneficial in one environment, and its loss enables adaption to another environment.

### 12.3 Germ line/soma distinction

The differentiation of the germ line and the soma is a particularly important concept in aging research (Monaghan and Metcalfe, 2019). The germ line cells express high levels of the enzyme telomerase, which replaces the telomere sequences lost during cell division. Because of this and other efficient genome maintenance mechanisms, the germ line cell lineage is essentially immortal, as long as the species does not go extinct (Raz and Yamashita, 2021). In contrast, normal somatic cells have limited replication potential and are mortal, sometimes called the “disposable soma” (Kirkwood, 2005). The soma undergoes aging (senescence) due to the genetic mechanisms of antagonistic pleiotropy and sexual antagonistic pleiotropy, leading to trade-offs between growth, differentiation, sexual differentiation and reproduction *versus* life span (Hughes and Reynolds, 2005; Tower, 2006; Waskar et al., 2009; Carter and Nguyen, 2011; Byars and Voskarides, 2020; Tower et al., 2020). The germ line cells give rise to egg and sperm, and these cells are passed on to the progeny. In contrast, the somatic cells are not passed on to the progeny. It is possible to consider this mechanism in terms of an AB replicator model, where instability of the B subunit prevents its inheritance. If germ line cells are considered as the stable replicator subunit (A), and the somatic cells as the unstable subunit (B), this corresponds to models where A can re-create B (Figure 4). One reason it may be selectively advantageous to make the somatic cells unstable relative to the germ line, is to prevent the transmission of damaged somatic cell genomes to the progeny. Indeed, it has been suggested that the evolution of the germ line/soma distinction might be particularly important in preventing the transmission of damaged somatic cell mitochondrial genomes to the progeny (Radzvilavicius et al., 2016). This might be interpreted in terms of model 3, elimination of damage (Figure 4C), where the somatic cells represent the damaged B subunits.

### 12.4 Grandmother hypothesis

The possible reasons for the evolution of the human female menopause have long been a popular topic for research and conjecture (Kirkwood and Shanley, 2010). One possibility is that the declining force of natural selection with age has allowed for the fixation of an allele or alleles in humans that causes menopause. An alternative explanation is the Grandmother hypothesis, which posits that there can be active selection for the menopause in species where there is significant inter-generational caregiving by females (Hawkes et al., 1998). The idea is that the menopause enables post-reproductive females to focus their resources and caregiving on existing offspring and grand-offspring. As long as the benefit to overall reproductive fitness of the lineage outweighs the cost of the female producing no further progeny at late ages, then the menopause can be selectively advantageous. Consistent with the Grandmother hypothesis, female reproductive cessation and post-reproductive caregiving has now been observed in multiple species, including humans and killer whales (Hawkes and Coxworth, 2013; Croft et al., 2017; Grimes et al., 2023; Nielsen et al., 2023). It is possible to consider this mechanism in terms of an AB replicator model. If female germ line cells are considered as the unstable replicator subunit (B), and the somatic cells as the stable subunit (A), this corresponds to model 1, where free A subunits are beneficial by increasing the activity of other replicators (Figure 3A). Notably, in this Grandmother hypothesis model, at the level of the individual female replicator, the germ line is the unstable B subunit, By contrast, in the above model for inheritance, the somatic cells are the unstable B subunit. Indeed, it is possible and expected that more than one SAI mechanism may be functioning at the same time.

### 12.5 Social networks

Social networks in humans and other organisms can emerge and change through time in ways that might be related to SAI (Hobson et al., 2013; Hilbert et al., 2016). The *de novo* emergence of a social network is reported to involve dynamic formation and breakage of links (Pomeroy et al., 2020; Xu et al., 2021). Online social networks have been proposed to display self-organizing criticality and the emergence of new functional properties (Tadic et al., 2017; Tadic and Melnik, 2021). By contrast, the potential role of component instability in the possible replication and proliferation of social networks is less explored, and this may be an interesting area for future research.

## 13 Concluding remarks

The examples of SAI discussed here include both well-studied and hypothetical mechanisms, and SAI appears to be common in cells, pre-biotic systems and in replicator computer simulations. It was generally possible to assign these examples of SAI to one or more of three underlying models, where the models describe benefits in addition to the generation of energy or mobilization of building blocks. Given the apparent requirement for proteases and nuclease in minimal cells, it appears reasonable to suggest that SAI by degradation is an emergent property of multi-subunit replicators, and is essential for life. The further design of synthetic replicators and synthetic cells may benefit from inclusion of components with SAI. For example, creation of the elusive RNA polymerase ribozyme self-replicator might by facilitated by inclusion of a subunit with SAI.

Limitations of the discussion include the broad definitions of SAI and the proposed models. On the one hand, broad definitions are needed to encompass the various examples of SAI presented, and are consistent with a mechanism that might extend to all living systems. On the other hand, overly-broad definitions increase the chances of including examples that might only superficially resemble any significant underlying mechanisms of SAI. Another limitation of the discussion is in regard to gene sequence changes. Models for how SAI can maintain genetic diversity are explored, but whether gene sequence change might be considered a form of SAI is generally not addressed. Through evolution, the most highly conserved genes include the ones encoding ribosomal RNAs, tRNAs, ribosomal proteins, translation initiation factors, ABC transporters and RNA polymerase (Harris et al., 2003; Isenbarger et al., 2008; Srikant, 2020). Conceivably, the relationship between these highly-conserved genes and the more rapidly evolving genes could be modeled in terms of SAI.

In the future, it may be useful to further explore SAI through additional comparative studies, computer modeling, and further testing of the models using synthetic replicators and minimal cells.
